# Aberrant tau accumulation caused by *MAPT* mutations induces early pathological changes in axonal transport that are rescued by p38α inhibition

**DOI:** 10.1038/s41593-026-02266-4

**Published:** 2026-06-04

**Authors:** Edoardo Moretto, Anna Masato, Chiara Panzi, André T. Lopes, Skye Stuart, Samantha De La-Rocque, Maria Giuseppa Caso, Ian J. White, Samuel S. Harris, Marc Aurel Busche, Giampietro Schiavo

**Affiliations:** 1 UK Dementia Research Institute at University College London, London, UK; 2 Department of Neuromuscular Diseases, UCL Queen Square Motor Neuron Disease Centre, UCL Queen Square Institute of Neurology, University College London, London, UK; 3 Institute of Neuroscience, CNR, Milan, Italy; 4 Laboratory for Molecular Cell Biology, University College London, London, UK; 5 Department of Biomedicine, University of Basel, Basel, Switzerland; 6 University Hospital of Geriatric Medicine Felix Platter, University of Basel, Basel, Switzerland; 7 Present Address: Institute of Neuroscience, CNR, Milan, Italy

**Keywords:** Cellular neuroscience, Dementia, Molecular neuroscience, Membrane trafficking

## Abstract

Impairments in axonal transport have been implicated in the pathogenesis of tauopathies, including frontotemporal dementia and Alzheimer’s disease, yet the underlying mechanisms and reversibility of these deficits are largely unknown. In particular, the impacts of tau mutations, phosphorylation and aggregation on axonal transport in vivo remain controversial. By using two-photon imaging of axonal transport of BDNF granules in the mouse cortex, we reveal that deficits in axonal transport arise in vivo at early stages of tau pathology, preceding tangle formation and neuronal death. Mechanistically, these impairments are caused by the enlargement of tau envelopes on microtubules, which act as functional barriers for transport. Crucially, these deficits are reversed by inhibiting MAPK p38α. Together, our work demonstrates that tau pathology causes reversible deficits in axonal transport in vivo, posing the basis for pharmacological interventions to restore the physiological flux of axonal organelles and cargoes in tauopathies.

## Abstract (web summary)

Imaging axonal transport in vivo in the mouse cortex reveals that deficits in axonal transport arise at early stages of tau pathology, are caused by enlarged tau envelopes and are reversed by inhibiting MAPK p38α.

## Main

Tau is abundantly expressed in neurons, and its aggregation is regarded as a major driver of neuronal dysfunction and death in neurodegenerative diseases termed tauopathies, which include Alzheimer’s disease (AD) and frontotemporal dementia (FTD)^1^, where its progressive spread throughout the brain strongly correlates with cognitive decline^2,3^. Mutations in the gene encoding tau (*MAPT*) are causative of certain tauopathies^1,4^ and promote tau aggregation^1^, which is believed to initiate in axons^5,6^. Tau exerts several physiological functions, including axonal microtubule binding and stabilization^1^. Therefore, it is not surprising that tau regulates axonal transport in cultured neurons under both physiological and pathological conditions^1,7^. Crucially, however, the precise role of tau in microtubule stabilization and axonal transport in vivo remains unclear^8–11^.

Axonal transport is the process through which neurons shuttle organelles, including lysosomes and secretory granules, as well as mRNAs and protein complexes, between the cell body and axon terminals. This process is fundamental for neuronal differentiation, function and survival as highlighted by the large number of genes encoding axonal transport components found mutated in neurological disorders^12,13^. Axonal transport restoration in animal models of hereditary peripheral neuropathy and amyotrophic lateral sclerosis improves disease symptoms, suggesting a central role for this process in pathogenesis^14–16^.

To uncover the role of axonal transport defects in tauopathies, we established an in vivo assay that uses two-photon (2P) imaging to monitor axonal transport of brain-derived neurotrophic factor (BDNF)-containing secretory granules in vivo, achieving single-organelle resolution. We revealed that FTD P301S/P301L mutant tau impairs axonal transport at very early stages of pathology in a tauopathy mouse model, before the appearance of tangles and neuronal death. We also demonstrated that these deficits are reversible, as pharmacological inhibition of the mitogen-activated protein kinases (MAPKs) p38α/p38β restored axonal transport deficits during disease progression. Interestingly, p38α acts as a master kinase for tau, triggering further phosphorylation by other kinases^17,18^, and p38 is overactivated in neurons of individuals affected by tauopathies^19–22^. In turn, p38 inhibition or its genetic ablation has shown beneficial effects in AD^23–26^ and FTD^27^ models.

We found that axonal transport deficits were associated with aberrantly large clusters of tau in axons, which are reminiscent of tau envelopes^28–30^. These structures were first identified using in vitro polymerized microtubules and recombinant tau and are formed via the cooperative binding of multiple tau molecules to microtubules^28–30^. Tau envelopes acted as obstacles for axonal transport in a reconstituted system^28–30^, yet their existence in cultured neurons and in the intact nervous system is still unclear. Here, we found that p38α/p38β inhibition decreases mutant tau phosphorylation and reduces the size of tau envelopes, counteracting the detrimental effects of FTD P301S/P301L mutant tau on axonal transport both in vitro and in vivo.

## Results

### In vivo imaging of axonal transport of BDNF^+^ vesicles in the mouse brain

**Fig. 1:**
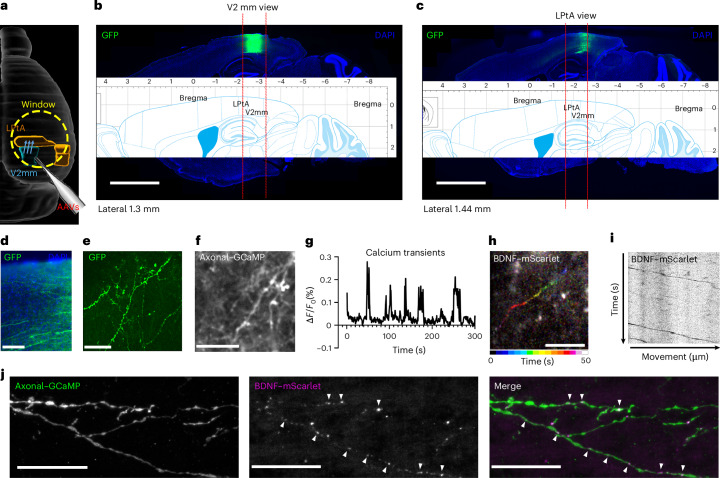
In vivo imaging of axonal transport of BDNF^+^ vesicles in the mouse brain. **a**, Schematic of the experimental strategy. AAVs, encoding different fluorescent reporters, were injected into the V2mm (blue). Neurons within this area project axons (blue arrows) to the LPtA (orange). A cranial window (yellow) was created during the same surgery over both areas. **b**, Representative sagittal section of a mouse brain injected using the protocol depicted in **a** with an AAV encoding cytosolic GFP and stained for DAPI. The superimposed atlas image (lateral 1.3 mm) shows correct targeting of the injection in the V2mm; *n* = 3 mice. **c**, Representative sagittal section of the same mouse brain as in **b** showing a more lateral view (lateral 1.44 mm). The superimposed atlas image shows that GFP^+^ axons reach the LPtA; *n* = 3 mice. **d**, Higher-magnification image of the LPtA from **c** showing GFP^+^ axons; scale bar, 100 µm; *n* = 3 mice. **e**, In vivo 2P imaging of GFP^+^ axons in the LPtA of a mouse injected as described in **a** with an AAV encoding cytosolic GFP; scale bar, 50 µm; *n* = 3 mice. **f**, In vivo 2P imaging of axonal–GCaMP6^+^ axons in the LPtA of a mouse injected with the AAV hSyn-axonal-GCaMP6 as described in **a**; scale bar, 20 µm; *n* = 3 mice. **g**, Representative trace of the variation in axonal–GCaMP6 intensity (% Δ*F*/*F*_0_) from a region of interest (ROI) from **f** over 5 min of recording. **h**, Temporal color-coded projection of 50 s of recording of BDNF–mScarlet granule movement in the LPtA of a mouse injected with the AAV CamKIIα-BDNF-mScarlet as described in **a**; scale bar, 20 µm. **i**, Kymograph showing movement of BDNF–mScarlet transport granules in an axon recorded in vivo. **j**, Confocal image of an axon double positive for axonal–GCaMP6 and BDNF–mScarlet imaged in a brain slice obtained from a mouse injected as described in **a** with both AAVs and transcardially perfused and stained for GCaMP6 and mScarlet; *n* = 3 mice. Arrowheads indicate mScarlet^+^ granules; scale bars, 20 µm.

Although the impact of tau pathology on axonal transport in cultured neurons is well established, in vivo experiments have led to conflicting results^8–11^. These experiments were, however, performed in nerve tracts less affected by tau pathology in human disease and by monitoring axonal transport with limited spatiotemporal resolution^8–11^. To address these limitations, we established a method for axonal transport analysis with far greater spatiotemporal resolution that we applied to brain regions substantially affected in tauopathies such as the association and parietal cortices^31,32^. This method involved the implantation of a cranial optical window obtained by substituting a portion of the skull with a glass coverslip. During the surgery, we injected adeno-associated viruses (AAVs) encoding an axonally targeted genetic calcium indicator (axonal–GCaMP6) and fluorescent organelle markers (Fig. 1). We targeted the secondary visual cortex (V2mm), whose neurons project axons to the lateral parietal association cortex (LPtA; Fig. 1a)^33^, a region that integrates inputs from different sensory areas^34^.

Tests were conducted in wild-type (WT) C57BL/6 mice by injecting an AAV encoding cytosolic green fluorescent protein (GFP) under the *Camk2a* promoter to target excitatory neurons, which are the most affected in tauopathies^35^. Immunocytochemistry showed the correct targeting of the V2mm (Fig. 1b) and the presence of GFP^+^ axons in the LPtA (Fig. 1c,d), which were also visible via 2P imaging in a living mouse (Fig. 1e). Axonal–GCaMP6^36^ was used to monitor calcium transients as a proxy for neuronal activity (Fig. 1f,g). To assess axonal transport, we focused on BDNF, which is sorted to secretory granules and plays crucial roles in the central nervous system, including regulating synaptic transmission and plasticity and thus cognitive function^37^. These organelles are generated in the soma and travel anterogradely through the axon to reach synapses^38–40^. Because tau regulates mostly anterograde transport due to the competition of tau and kinesins^41–43^, the anterograde bias of BDNF-containing vesicles made them ideal to study tau pathology-induced impairments.

Thus, we designed an AAV encoding BDNF–mScarlet to visualize individual BDNF-containing organelles traveling along axons with a 0.6-s temporal resolution (Fig. 1h,i and Supplementary Videos 1 and 2). Immunohistochemistry confirmed that axonal–GCaMP6 and BDNF–mScarlet were present in axons (Fig. 1j). We have thus established a reliable method for analyzing axonal transport in vivo in brain regions affected in human disease with enhanced spatiotemporal resolution.

### Axonal transport is impaired in rTg4510 mice and is restored by p38α/p38β inhibition at early stages of disease

We monitored axonal transport of BDNF-containing granules in the rTg4510 tauopathy mouse model. In this strain, the expression of FTD-linked human P301L mutant tau is restricted to excitatory neurons^44,45^ and reduced by doxycycline treatment; therefore, doxycycline-fed rTg4510 animals allowed us to control for effects of other unrelated genes^46^.

**Fig. 2:**
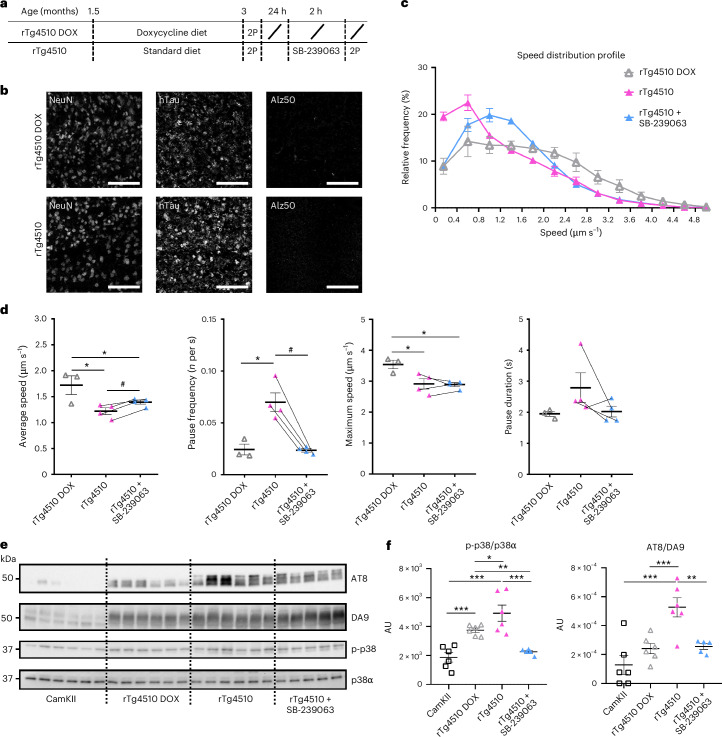
Axonal transport is impaired in rTg4510 mice and is restored by p38α/p38β inhibition at early stages of disease. **a**, Experimental timeline. rTg4510 mice were fed either a standard diet or a diet supplemented with doxycycline (200 mg per kg (body weight)) for 6 weeks (rTg4510 DOX; gray). Mice were injected with AAVs encoding axonal–GCaMP6 and BDNF–mScarlet in the V2mm area and implanted with a cranial window as described in Fig. 1. rTg4510 mice were then imaged by 2P microscopy at 3 months of age under light anesthesia. The mice maintained on a standard diet were injected intraperitoneally with vehicle, imaged once (rTg4510, magenta), left to recover for 24 h, injected intraperitoneally with SB-239063 (100 mg per kg (body weight)) and imaged after 2 h under light anesthesia (rTg4510 + SB-239063; blue). **b**, Confocal images of coronal slices obtained from rTg4510 mice treated with doxycycline or not and stained for NeuN to mark all neurons, hTau and Alz50 to detect aggregated tau; scale bars, 100 µm. **c**, Speed distribution profiles of anterograde axonal transport of BDNF–mScarlet-containing vesicles; *n* = 3 mice for rTg4510 DOX and 4 mice for rTg4510 and rTg4510 + SB-239063. **d**, Quantification relative to **a**–**c**. Ninety randomly selected tracks were analyzed per mouse to determine the average speed (one-way analysis of variance (ANOVA) *F* = 6.628, *P* = 0.0201, Newman–Keuls multiple comparisons test; paired two-tailed Student’s *t*-test rTg4510 versus rTg4510 + SB-239063, *P* = 0.0125), frequency of pausing (one-way ANOVA *F* = 18.93, *P* = 0.0009, Newman–Keuls multiple comparisons test; paired two-tailed Student’s *t*-test rTg4510 versus rTg4510 + SB-239063, *P* = 0.0204), maximum speed (one-way ANOVA *F* = 7.315, *P* = 0.0156, Newman–Keuls multiple comparisons test; paired two-tailed Student’s *t*-test rTg4510 versus rTg4510 + SB-239063, *P* = 0.8814) and duration of pauses (Kruskal–Wallis test *P* = 0.1060; paired two-tailed Wilcoxon matched-pairs signed-rank test rTg4510 versus rTg4510 + SB-239063, *P* = 0.25); *n* = 3 mice for rTg4510 DOX and 4 mice for rTg4510 and rTg4510 + SB-239063; all animals were female. **e**, Western blots of forebrain lysates obtained from a matching cohort of transactivator-only (CamKII) or rTg4510 3-month-old mice fed a standard diet (rTg4510) or a diet supplemented with doxycycline for 6 weeks (rTg4510 DOX) or injected intraperitoneally with SB-239063 (100 mg per kg (body weight); rTg4510 + SB-239063) 2 h before culling. All other animals received an intraperitoneal injection of the vehicle 2 h before culling. Blots were probed for p38α, phosphorylated p38 (p-p38), total tau (DA9) and AT8 (S202/T205 p-tau). **f**, Quantification relative to **e** of p38 activation (p-p38/p38α ratio; one-way ANOVA *F* = 16.57, *P* < 0.0001, two-stage linear step-up procedure of Benjamini, Krieger and Yekutieli multiple comparisons; CamKII versus rTg4510 DOX *P* = 0.001; CamKII versus rTg4510 *P* ≤ 0.0001; CamKII versus rTg4510 + SB-239063 *P* = 0.4544; rTg4510 DOX versus rTg4510 *P* = 0.0246; rTg4510 DOX versus rTg4510 + SB-239063 *P* = 0.0082; rTg4510 versus rTg4510 + SB-239063 *P* < 0.0001) and tau phosphorylation (AT8/DA9; one-way ANOVA *F* = 10.71, *P* = 0.0002, two-stage linear step-up procedure of Benjamini, Krieger and Yekutieli multiple comparisons; CamKII versus rTg4510 DOX *P* = 0.1392; CamKII versus rTg4510 *P* ≤ 0.0001; CamKII versus rTg4510 + SB-239063 *P* = 0.1129; rTg4510 DOX versus rTg4510 *P* = 0.0009; rTg4510 DOX versus rTg4510 + SB-239063 *P* = 0.851; rTg4510 versus rTg4510 + SB-239063 *P* = 0.0022). Each lane was normalized by the total protein content as visualized by Coomassie staining; *n* = 6 mice (3 males and 3 females) for CamKII, rTg4510 and rTg4510 DOX and *n* = 5 mice (3 males and 2 females) for rTg4510 + SB-239063. Bars represent the mean ± s.e.m. In **d**, the asterisks (*) indicate statistical significance as determined by one-way ANOVA with a post hoc correction for multiple comparisons (Newman–Keuls): *P < 0.05. The number symbol (^#^) indicates statistical significance as determined by paired two-tailed *t*-test between rTg4510 before and after injection with SB-239063; ^#^*P* < 0.05. Lines in **d** connect the same animal. In **f**, the asterisks (*) indicate statistical significance as determined by one-way ANOVA with a post hoc correction for multiple comparisons (false discovery rate, two-stage step-up method Benjamini, Krieger and Yekutieli); *P < 0.05, **P < 0.01 and ***P < 0.001; AU, arbitrary units. Source data

**Extended Data Fig. 1:**
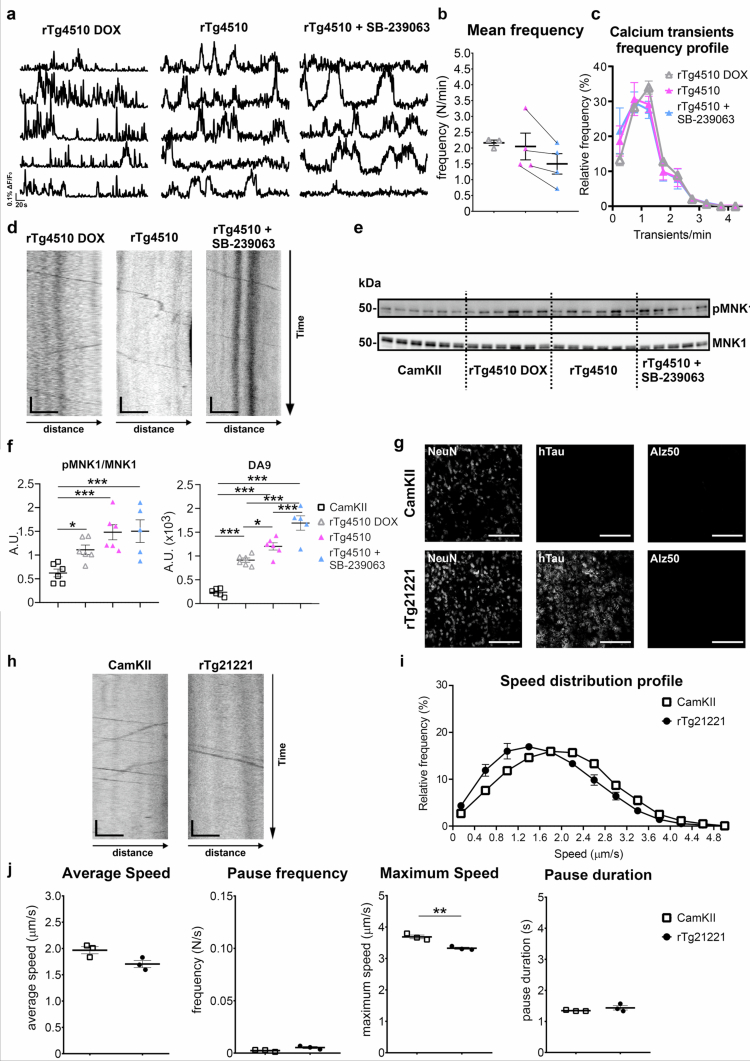
Axonal transport is impaired in rTg4510 mice and is restored by p38α/β inhibition at early stages of disease. Related to Fig. 2. **a)** The same groups of mice analysed in Fig. 2a-d were also monitored for axonal-GCaMP6 signal over 5 min under light anaesthesia. Tracks show spontaneous calcium transients of five randomly selected ROIs for each group. **b)** Mean frequencies of calcium transients for the three groups imaged in Fig. 2. n = 3 mice for rTg4510 DOX, 4 mice for rTg4510 and rTg4510 + SB-239063. (One-way ANOVA F = 1.084, p value = 0.3831, Newman-Keuls multiple correction; paired two-tailed Student’s t-test rTg4510 vs rTg4510 + SB-23906 p value = 0.0975). **c)** Frequency distribution of calcium transients in all recorded ROIs in the three groups. n = 3 mice for rTg4510 DOX, 4 mice for rTg4510 and rTg4510 + SB-239063 **d)** Representative kymographs of axonal trafficking of BDNF-mScarlet relative to Fig. 2a-d. Scale bar: vertical = 20 s; horizontal = 10 μm. **e)** The same samples from Fig. 2e-f were also blotted for pMNK1 and MNK1. **f)** Quantification relative to panel **e** of pMNK1/MNK1 (One-way ANOVA F = 7.83, p value = 0.0013, Two-stage linear step-up procedure of Benjamini, Krieger and Yekutieli multiple comparison, adj p values: CamKII vs rTg4510 DOX = 0.0272; CamKII vs rTg4510 = 0.0005; CamKII vs rTg4510 + SB-239063 = 0.0006; rTg4510 DOX vs rTg4510 = 0.0874; rTg4510 DOX vs rTg4510 + SB-239063 = 0.0846; rTg4510 vs rTg4510 + SB-239063 = 0.9202) and to Fig. 2e of DA9 (One-way ANOVA F = 54.17, p value < 0.0001, Two-stage linear step-up procedure of Benjamini, Krieger and Yekutieli multiple comparison, adj p values: CamKII vs rTg4510 DOX < 0.0001; CamKII vs rTg4510 < 0.0001; CamKII vs rTg4510 + SB-239063 < 0.0001; rTg4510 DOX vs rTg4510 = 0.0190; rTg4510 DOX vs rTg4510 + SB-239063 < 0.0001; rTg4510 vs rTg4510 + SB-239063 = 0.0005). Each lane was normalised on the total protein content as visualised by Coomassie staining. n = 6 mice (3 males and 3 females) for CamKII, rTg4510 and rTg4510 DOX and n = 5 mice (3 males and 2 females) for rTg4510 + SB-239063. **g)** Confocal images of coronal slices obtained from the transactivator only mice (CamKII) and from rTg21221 mice stained for NeuN, hTau and Alz50. Scale bar = 100 µm. **h)** Representative kymographs of axonal trafficking of BDNF-mScarlet from *in vivo* imaging of CamKII and rTg21221 mice. **i)** Frequency distribution of the instantaneous axonal speed of BDNF-mScarlet granules from *in vivo* imaging of CamKII (hollow black squares) and rTg21221 (black circles) mice. n = 3 mice per genotype, mice were in a male/female ratio of 2/1 **j)** Quantification of the average speed (µm/s) (Unpaired two-tailed Student’s t-test p value = 0.0523), frequency of pausing (N/s) (Unpaired two-tailed Student’s t-test p value = 0.0556), maximum speed (µm/s) (Unpaired two-tailed Student’s t-test p value = 0.0057) and pause duration (s) (Unpaired two-tailed Mann Whitney test p value = 0.4) of 90 randomly selected tracks of transport of BDNF-mScarlet granules per mouse. n = 3 mice per genotype, mice were in a male/female ratio of 2/1. Bars represent the mean ± SEM. Lines in **b** connect the same animal. In panel **f**, * indicate statistical significance in one-way ANOVA with post-hoc correction for multiple comparison (FDR, two-stage step-up method BKY): * = p < 0.05; ** = p < 0.01; *** = p < 0.001. In panel **j**, * indicates statistical significance in unpaired two-sided Student’s t-test. ** = p < 0.01. Source data

Based on the role of p38α/p38β both in tau phosphorylation^17,18^ and axonal transport modulation^14^, we tested the p38α/p38β inhibitor SB-239063 (Fig. 2a–d). rTg4510 mice were imaged first and then again 24 h later after administering SB-239063 (Fig. 2a)^47^. Experiments were performed on 3-month-old rTg4510 mice, which show expression of human tau (hTau) but little or no aggregation (Fig. 2b)^44^, modeling an early stage of pathology. Axonal calcium transient frequency was unaffected (Extended Data Fig. 1a–c), suggesting normal baseline neuronal activity.

The analysis of axonal transport of BDNF-containing vesicles showed that rTg4510 mice had an overall reduced transport speed, as well as a marked increase in the frequency of pausing events, compared to doxycycline-fed isogenic littermates (Fig. 2c,d and Extended Data Fig. 1d). Treatment with the p38α/p38β inhibitor rescued the pausing frequency and promoted a partial normalization of the transport speed (Fig. 2c,d), demonstrating the reversibility of these deficits. Analysis of forebrain lysates revealed that rTg4510 mice have overactive p38α and high levels of tau phosphorylation. Inhibition of p38α/p38β reverted both effects (Fig. 2e,f). MNK1, a kinase downstream of p38α^48^, is also overactivated in rTg4510 mice but appears unaffected by p38α inhibition (Extended Data Fig. 1e,f).

Because rTg4510 mice express high levels of hTau^44,45^, it was imperative to ascertain that the axonal transport impairment was specifically caused by tau mutation. We used the rTg21221 strain, which expresses WT hTau at levels similar to rTg4510 mice^49^ without any tau pathology nor behavioral changes^50^. rTg21221 animals showed hTau expression but little to no differences in axonal transport compared to littermates carrying only the CamKIIα-tet transactivator, being comparable to doxycycline-fed rTg4510 mice (Extended Data Fig. 1g–j). These results further confirmed that the axonal transport defects observed in rTg4510 mice are specifically driven by the tau mutation.

Our findings demonstrate that the axonal transport of BDNF-containing secretory granules is defective in the cerebral cortex of a tauopathy model at very early stages of pathology. These impairments are strictly caused by the expression of FTD-linked mutant tau and can be at least partially normalized by acute p38α/p38β inhibition.

### p38α/p38β inhibition enhances axonal transport in rTg4510 mice at later stages of disease

**Fig. 3:**
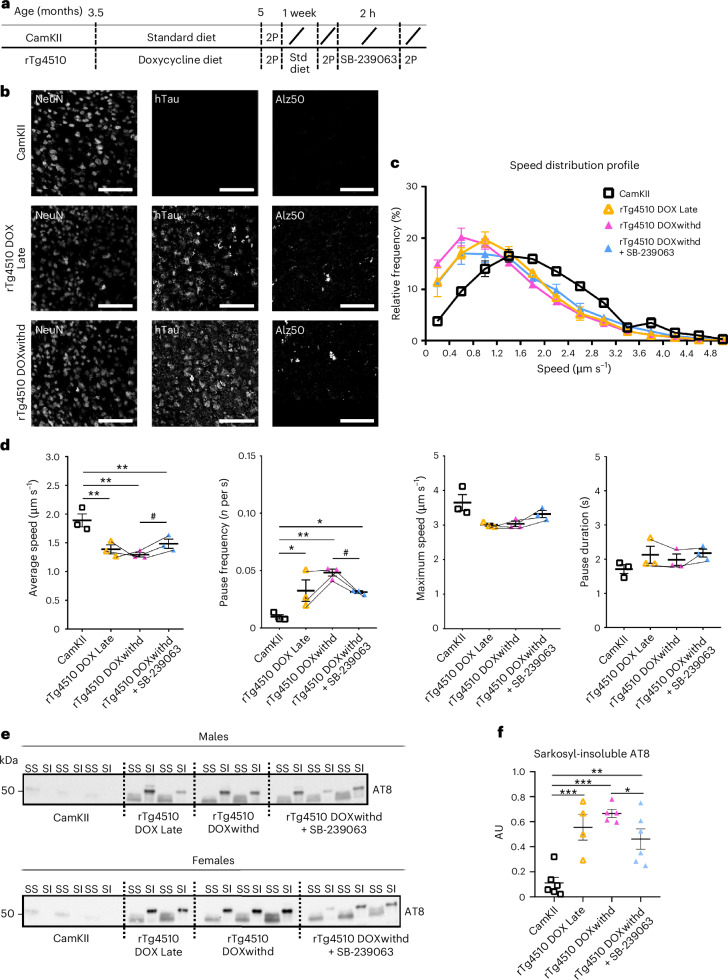
Acute inhibition of p38α/p38β enhances axonal transport in vivo in rTg4510 mice at later stages of disease. **a**, Experimental timeline. Mice were injected with AAVs encoding axonal–GCaMP6 and BDNF–mScarlet in the V2mm area, as described in Fig. 1. rTg4510 mice were fed doxycycline for 6 weeks from 3.5 months of age, injected intraperitoneally with vehicle and imaged once (rTg4510 DOX Late, orange). Subsequently, the doxycycline diet was withdrawn for 10 days, and animals were injected intraperitoneally with vehicle and reimaged (rTg4510 DOXwithd, magenta). Mice were then left to recover for 24 h before being injected intraperitoneally with SB-239063 and reimaged 2 h later (rTg4510 DOXwithd + SB-239063, blue). Transactivator-only mice, injected intraperitoneally with vehicle, were used as controls (CamKII, hollow black squares); Std, standard. **b**, Confocal images of coronal slices obtained from CamKII or rTg4510 mice fed with doxycycline (rTg4510 DOX Late) and after doxycycline withdrawal (rTg4510 DOXwithd) and stained for NeuN, hTau and Alz50; scale bars, 100 µm. **c**, Frequency distribution of the instantaneous speed of the axonal transport of BDNF–mScarlet-containing vesicles obtained by in vivo imaging of the animals as per **a**; *n* = 3 mice per group (all mice were female). **d**, Quantification of the average speed (one-way ANOVA *F* = 10.18, *P* = 0.0042, Newman–Keuls multiple comparisons test; paired two-tailed Student’s *t*-test: rTg4510 DOX Late versus rTg4510 DOXwithd, *P* = 0.4871; rTg4510 DOXwithd versus rTg4510 DOXwithd + SB-239063, *P* = 0.0412), frequency of pausing (one-way ANOVA *F* = 9.772, *P* = 0.0047, Newman–Keuls multiple comparisons test; paired two-tailed Student’s *t*-test: rTg4510 DOX Late versus rTg4510 DOXwithd, *P* = 0.1679; rTg4510 DOXwithd versus rTg4510 DOXwithd + SB-239063, *P* = 0.0483), maximum speed (Kruskal–Wallis test *P* = 0.0179, Dunn’s multiple comparisons test adjusted *P* values: CamKII versus rTg4510 DOX Late *P* = 0.1887, CamKII versus rTg4510 DOXwithd *P* = 0.1045, CamKII versus rTg4510 DOXwithd + SB-239063 *P* > 0.9999, rTg4510 DOX Late versus rTg4510 DOXwithd *P* > 0.9999, rTg4510 DOX Late versus rTg4510 DOXwithd + SB-239063 *P* = 0.8462, rTg4510 DOXwithd versus rTg4510 DOXwithd + SB-239063 *P* = 0.5366; Wilcoxon matched-pairs signed-rank test: rTg4510 DOX Late versus rTg4510 DOXwithd *P* > 0.9999; rTg4510 DOXwithd versus rTg4510 DOXwithd + SB-239063 *P* = 0.25) and pause duration (Kruskal–Wallis *P* = 0.2167; Wilcoxon matched-pairs signed-rank test: rTg4510 DOX Late versus rTg4510 DOXwithd *P* = 0.25; rTg4510 DOXwithd versus rTg4510 DOXwithd + SB-239063 *P* = 0.25) of 90 randomly selected tracks of transport of BDNF–mScarlet-containing granules per mouse of the animals as per **a**; *n* = 3 mice per group (all mice were female). **e**, Western blots of the sarkosyl-soluble (SS) and sarkosyl-insoluble (SI) fractions obtained from the forebrains of a matching cohort of transactivator-only (CamKII) or rTg4510 5-month-old mice, fed with doxycycline for 6 weeks (rTg4510 DOX Late), after 1 week of withdrawal of the doxycycline diet (rTg4510 DOXwithd) and injected intraperitoneally with SB-239063 (100 mg per kg (body weight); rTg4510 DOXwithd + SB-239063) 2 h before culling. All other animals received an intraperitoneal injection of the vehicle 2 h before culling. Blots were probed for AT8 (S202/T205 p-tau). **f**, Quantification (relative to the data in **e**) of sarkosyl-insoluble tau normalized over the sum of sarkosyl-soluble and sarkosyl-insoluble AT8 tau (one-way ANOVA *F* = 13.39, *P* < 0.0001, two-stage linear step-up procedure of Benjamini, Krieger and Yekutieli multiple comparisons test *P* values: CamKII versus rTg4510 DOX Late *P* = 0.0003; CamKII versus rTg4510 DOXwithd *P* ≤ 0.0001; CamKII versus rTg4510 DOXwithd + SB-239063 = 0.0011; rTg4510 DOX Late versus rTg4510 DOXwithd *P* = 0.3015; rTg4510 DOX Late versus rTg4510 DOXwithd + SB-239063 *P* = 0.3591; rTg4510 DOX Late versus rTg4510 DOXwithd + SB-239063 *P* = 0.0432). Each lane was normalized on the total protein content as visualized by Coomassie staining; *n* = 6 CamKII mice (3 males and 3 females), 4 rTg4510 DOX Late mice (2 males and 2 female), 5 rTg4510 DOXwithd mice (2 males and 3 females) and 6 rTg4510 DOXwithd + SB-239063 mice. Bars represent the mean ± s.e.m. In **d**, asterisks (*) indicate statistical significance as determined by one-way ANOVA with a post hoc correction for multiple comparisons (Newman–Keuls): *P < 0.05 and **P < 0.01. The number sign (^#^) indicates statistical significance as determined by paired two-sided Student’s *t*-test between animals before and after injection with SB-239063: ^#^*P* < 0.05. Lines in **d** connect the same animal. In **f**, asterisks (*) indicate statistical significance as determined by one-way ANOVA with a post hoc correction for multiple comparisons (false discovery rate, two-stage step-up method Benjamini, Krieger and Yekutieli): *P < 0.05, **P < 0.01 and ***P < 0.001. Source data

**Extended Data Fig. 2:**
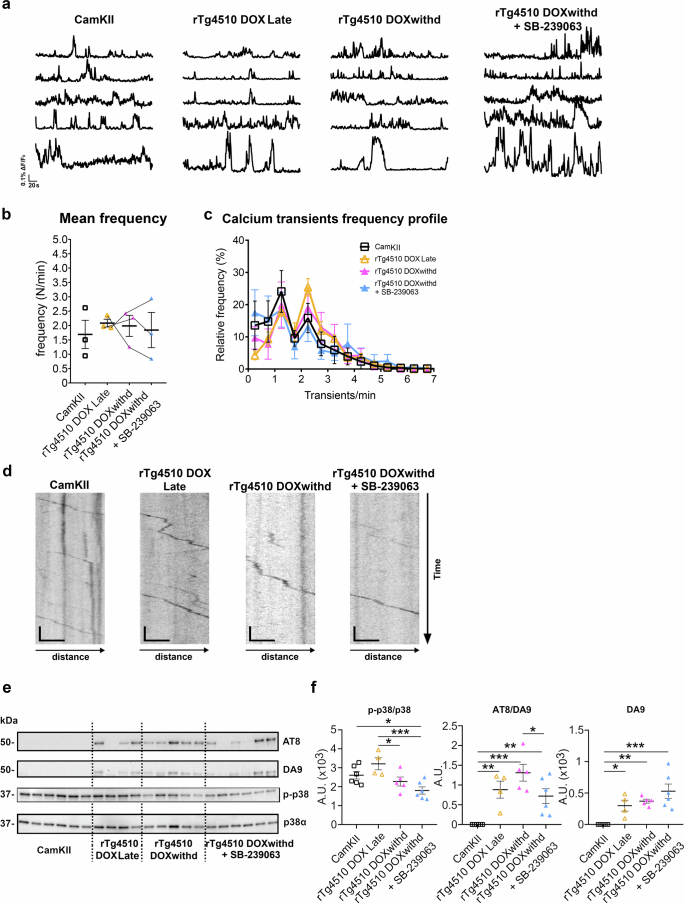
Acute inhibition of p38α/β enhances axonal transport in vivo in rTg4510 mice at later stages of disease. Related to Fig. 3. **a)** The same groups analysed in Fig. 3a-d were also monitored for axonal-GCaMP6 signal over 5 min under light anaesthesia. Tracks show spontaneous calcium transients of five randomly selected ROIs for each group. **b)** Mean frequencies of calcium transients for the groups imaged in Fig. 3. (One-way ANOVA F = 0.1550, p value = 0.9236, Newman-Keuls multiple comparison. Paired two-tailed Student’s t-test p values = rTg4510 DOX Late vs rTg4510 DOXwithd = 0.8625; rTg4510 DOXwithd vs rTg4510 DOXwithd + SB-239063 = 0.7097). n = 3 mice per group, all mice were female. **c)** Frequency distribution of calcium transients from all recorded ROIs in the groups imaged in Fig. 3. n = 3 mice per genotype, all animals were female. **d)** Representative kymographs of axonal trafficking of BDNF-mScarlet relative to Fig. 3a-d. Scale bar: vertical = 20 s; horizontal = 10 μm. **e)** Western blots of forebrain lysates obtained from transactivator only (CamKII) or rTg4510 5-month-old mice, fed with doxycycline for 6 weeks (rTg4510 DOX Late), after 1 week of withdrawal of the doxycycline diet (rTg4510 DOXwithd) and injected intraperitoneally with SB-239063 (100 mg/Kg) (rTg4510 DOXwithd + SB-239063) 2 h before culling. All other animals received an intraperitoneal injection of the vehicle 2 h before culling. Blots were probed for p38α, phosphorylated p38 (p-p38), total tau (DA9), and AT8 (S202/T205 p-Tau). **f)** Quantification relative to panel **e** of p38 activation (p-p38/p38α ratio) (One-way ANOVA F = 6.287, p value 0.0046 Two-stage linear step-up procedure of Benjamini, Krieger and Yekutieli multiple comparison p values: CamKII vs rTg4510 DOX Late = 0.0924; CamKII vs rTg4510 DOXwithd = 0.3047; CamKII vs rTg4510 DOXwithd + SB-239063 = 0.0154; rTg4510 DOX Late vs rTg4510 DOXwithd = 0.0161; rTg4510 DOX Late vs rTg4510 DOXwithd + SB-239063 = 0.0006; rTg4510 DOXwithd vs rTg4520 DOXwithd + SB-239063 = 0.1497), tau hyperphosphorylation (AT8/DA9) (One-way ANOVA F = 11.06, p value 0.0003 Two-stage linear step-up procedure of Benjamini, Krieger and Yekutieli multiple comparison p values: CamKII vs rTg4510 DOX Late = 0.0025; CamKII vs rTg4510 DOXwithd < 0.0001; CamKII vs rTg4510 DOXwithd + SB-239063 = 0.0049; rTg4510 DOX Late vs rTg4510 DOXwithd = 0.1160; rTg4510 DOX Late vs rTg4510 DOXwithd + SB-239063 = 0.5199; rTg4510 DOXwithd vs rTg4510 DOXwithd + SB-239063 = 0.0213) and total tau (DA9) (One-way ANOVA F = 10.00, p value 0.0005 Two-stage linear step-up procedure of Benjamini, Krieger and Yekutieli multiple comparison p values: CamKII vs rTg4510 DOX Late = 0.0151; CamKII vs rTg4510 DOXwithd = 0.0024; CamKII vs rTg4510 DOXwithd + SB-239063 < 0.0001; rTg4510 DOX Late vs rTg4510 DOXwithd = 0.5423; rTg4510 DOX Late vs rTg4510 DOXwithd + SB-239063 = 0.0524; rTg4510 DOXwithd vs rTg4520 DOXwithd + SB-239063 = 0.1434). Each lane was normalised on the total protein content as visualised by Coomassie staining. n = 6 CamKII mice (3 males and 3 females), 4 (2 males and 2 female) rTg4510 DOX Late, 5 (2 males and 3 females) rTg4510 DOXwithd and 6 (3 male and 3 female) rTg4510 DOXwithd + SB-239063. Bars represent the mean ± SEM. Lines in **b** connect the same animal. In panel **f**, * indicate statistical significance in one-way ANOVA with post-hoc correction for multiple comparison (FDR, two-stage step-up method BKY): * = p < 0.05; ** = p < 0.01; *** = p < 0.001. Source data

We next tested whether axonal transport defects are present and reversible at later stages of disease. We used 5-month-old rTg4510 mice, fed with doxycycline from 3.5 months of age for 6 weeks, which present cortical tau aggregates while expressing lower levels of hTau (Fig. 3a,b)^44^. As controls, we used littermates carrying the CamKIIα-tet transactivator only. As before, axonal calcium transients were indistinguishable between groups, suggesting normal baseline neuronal activity (Extended Data Fig. 2a–c).

Analysis of axonal transport showed that more advanced tau pathology also reduces the average speed of BDNF^+^ vesicles and increases pausing frequency (Fig. 3c,d and Extended Data Fig. 2d), similar to what was observed in younger mice (Fig. 2a–d). Although not statistically significant, doxycycline withdrawal induced further axonal transport impairment. rTg4510 mice were left to recover for 24 h and were then reimaged after SB-239063 administration (Fig. 3a). Importantly, acute p38α/p38β inhibition was able to partially rescue the axonal transport defects in these mice, demonstrating reversibility also in the presence of tangles (Fig. 3).

We further evaluated the aggregational state of tau by treating mouse forebrains with sarkosyl. AT8^+^ tau was increased in the sarkosyl-insoluble fraction of rTg4510 forebrains, an effect significantly reduced by p38α/p38β inhibition (Fig. 3e,f). Notably, doxycycline withdrawal for 1 week did not significantly alter sarkosyl-insoluble tau levels.

Analysis of forebrain homogenates showed that, as for the younger cohort, tau appeared hyperphosphorylated in rTg4510 mice, an effect reverted by the acute inhibition of p38α/p38β (Extended Data Fig. 2e,f). However, this kinase was not hyperactivated compared to CamKII-only littermates, suggesting that other factors are at play (Extended Data Fig. 2e,f). Similarly, withdrawal of doxycycline did not induce significant differences in tau phosphorylation levels.

### Sustained inhibition of p38α enhances axonal transport in vivo in rTg4510 mice

Given that acute inhibition of p38α/p38β showed an amelioration of the axonal transport deficits in rTg4510 mice, we hypothesized that a sustained inhibition of p38 could provide additional benefits. To test this, we chose neflamapimod, a compound with excellent oral bioavailability^51^ and 20-fold higher selectivity for p38α than p38β^52^. Neflamapimod previously showed a significant reduction in both phosphorylated and total tau in cerebrospinal fluid in an AD clinical trial^53^.

**Fig. 4:**
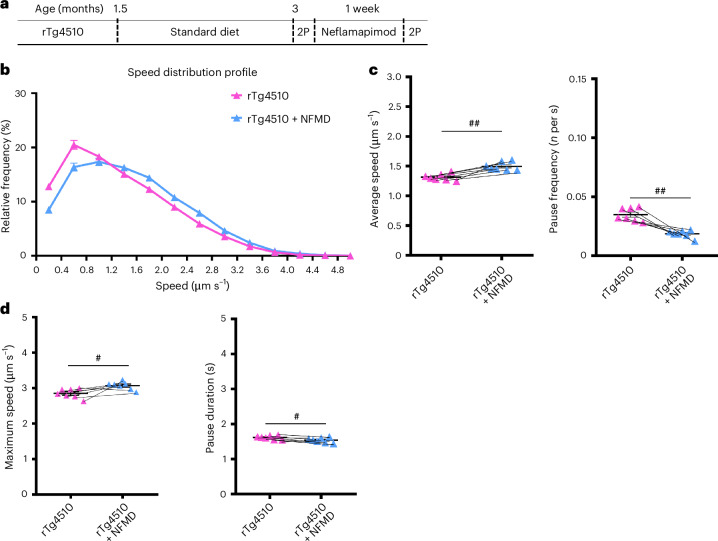
Sustained inhibition of p38α enhances axonal transport in vivo in rTg4510 mice. **a**, Experimental timeline. Mice were injected with AAVs encoding axonal–GCaMP6 and BDNF–mScarlet in the V2mm area, as described in Fig. 1. Three-month-old rTg4510 mice were imaged once (rTg4510, magenta), left to recover for 24 h and treated by oral gavage twice daily for 5 days with 3 mg per kg (body weight) neflamapimod. At the end of day 5 and around 2 h after the last administration, mice were imaged again (rTg4510 + NFMD, blue). **b**, Speed distribution profile of the axonal transport of BDNF–mScarlet-containing vesicles obtained by in vivo imaging. **c**, Quantification of the average speed (paired two-tailed Student’s *t*-test *P* = 0.0012) and frequency of pausing (paired two-tailed Student’s *t*-test *P* = 0.0016) of 90 randomly selected tracks of BDNF–mScarlet granules per mouse in rTg4510 mice before and after treatment with neflamapimod. **d**, Quantification of the maximum speed (paired two-tailed Student’s *t*-test *P* = 0.0294) and pause duration (paired two-tailed Student’s *t*-test *P* = 0.0221) of 90 randomly selected tracks of BDNF–mScarlet-containing granules per mouse in rTg4510 mice before and after treatment with neflamapimod; *n* = 7 mice (5 females and 2 males). Bars represent the mean ± s.e.m. Statistical significance was determined by paired two-sided Student’s *t*-tests between rTg4510 before and after treatment with neflamapimod: ^#^*P* < 0.05 and ^#^^#^*P* < 0.01. Lines in **c** and **d** connect the same animal. Source data

**Extended Data Fig. 3:**
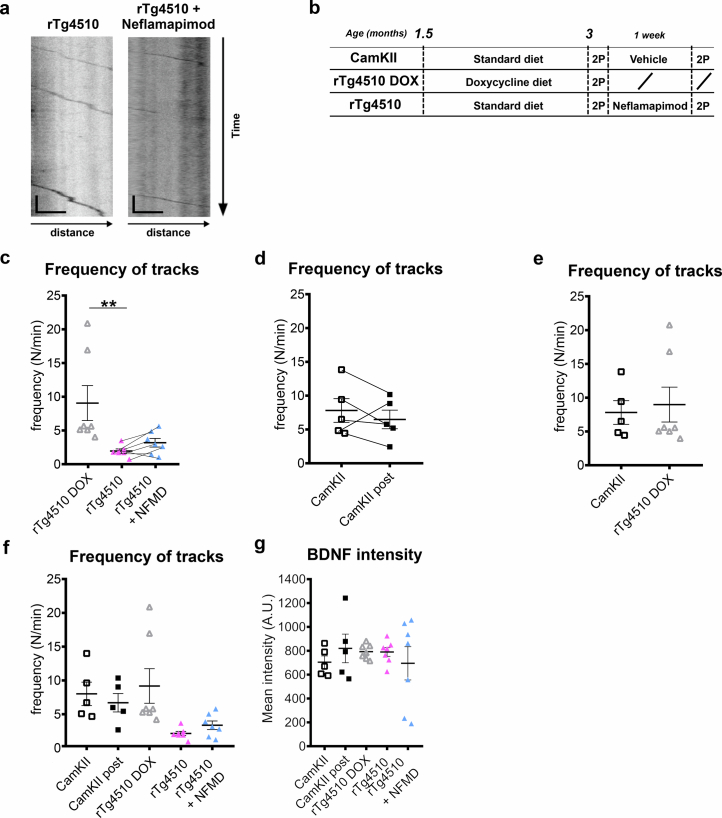
Sustained inhibition of p38α enhances axonal transport in vivo in rTg4510 mice. Related to Fig. 4. **a)** Representative kymographs of axonal trafficking of BDNF-mScarlet relative to Fig. 4a-d. Scale bar: vertical = 20 s; horizontal = 10 μm. **b)** Experimental timeline. Mice were injected with AAVs encoding for axonal-GCaMP6 and BDNF-mScarlet in the V2mm area as described in Fig. 1. CamKII- transactivator only mice were fed standard diet, imaged once (hollow squares, CamKII), left to recover for 24 h and then treated by oral gavage twice daily for five days with the vehicle of neflamapimod before being imaged again (filled squares, CamKII post). A group of 3-month-old rTg4510 mice were fed doxycycline (200 mg/Kg) for six weeks before being imaged (rTg4510 DOX, grey). Another group of rTg4510 were fed standard diet, imaged once (rTg4510, magenta), left to recover for 24 h and then treated by oral gavage twice daily for 5 days with 3 mg/Kg of neflamapimod. At the end of day 5 and around 2 h after the last administration, mice were imaged again (rTg4510+NFMD, blue). **c)** Automatic analysis of the frequency of BDNF-mScarlet moving organelles imaged over 10 min in rTg4510 mice fed with doxycycline (rTg4510 DOX, grey triangles), or not (rTg4510, magenta). rTg4510 were subjected to 5 days of treatment with neflamapimod as in Fig. 4 and then reimaged (rTg4510+NFMD, blue). Frequency of tracks (N/min). (Kruskal-Wallis test p value = 0.0004, Dunn’s multiple comparison adj p value: rTg4510 DOX vs rTg4510 = 0.0018, CamKII vs rTg4510 + NFMD = 0.0598, rTg4510 vs rTg4510 + NFMD = 0.8152; Wilcoxon matched-pairs signed rank test p value rTg4510 vs rTg4510 + NFMD = 0.0781). n = 7 mice per group. **d)** Automatic analysis of the frequency of BDNF-mScarlet moving organelles imaged over 10 min of recording in CamKII transactivator-only mice imaged before (CamKII, hollow black square) and after 5 days of oral gavage twice daily with vehicle (CamKII post, filled black square). (Paired two-tailed Student’s t-test p value CamKII vs CamKII post = 0.4173). n = 5 mice per group. **e)** Automatic analysis of the frequency of BDNF-mScarlet moving organelles imaged over 10 min of recording in transactivator only mice (CamKII, hollow black square) and doxycycline fed rTg4510 mice (rTg4510 DOX, grey). (unpaired Mann Whitney test p value CamKII vs rTg4510 DOX > 0.9999). **f)** Overall representation of all the frequencies of BDNF-mScarlet moving organelles over 10 min of recording for all groups analysed in Extended Data Fig. 4b-d. **g)** Quantification of the mean intensity of BDNF-mScarlet signal as measured in the first frame of *in vivo* imaging for all groups analysed in Extended Data Fig. 4b-d. (One-way ANOVA F = 0.4019 p value 0.8054). rTg4510 DOX were 4 females and 3 males; rTg4510 were 5 females and 2 males; CamKII mice were 4 females, 1 male. Bars represent the mean ± SEM. ** = p < 0.01: statistical significance in Kruskal-Wallis rank test with post-hoc correction for multiple comparison (Dunn’s). Lines in **c,d** connect the same animal. Source data

We explored the effect of neflamapimod on axonal transport of BDNF–mScarlet vesicles in 3-month-old rTg4510 mice by imaging them before and after treatment (Fig. 4a). We implemented a dosing previously used in independent animal studies^48^ and observed widespread enhancement of transport (Fig. 4b–d and Extended Data Fig. 3a). These data show that sustained p38α inhibition further improved transport dynamics compared to a single dosing, resulting in increased maximum speed and reduced pause duration, which were not altered by acute inhibition (compare Figs. 4c,d and 2d).

Next, we extended our study to additional control groups that we analyzed via an automated transport tracking workflow (Extended Data Fig. 3a,b,f and Methods). This approach showed reduced flux of BDNF-containing granules in rTg4510 mice compared to doxycycline-fed littermates, which was partially rescued by neflamapimod treatment, although without reaching statistical significance (Extended Data Fig. 3a–c). Importantly, doxycycline-fed rTg4510 mice showed transport levels comparable to CamKIIα-tet-only littermates (Extended Data Fig. 3d), and vehicle treatment had no effect on the latter group (Extended Data Fig. 3e). We also verified that the BDNF–mScarlet signal intensity was similar in all experimental groups (Extended Data Fig. 3g).

Together, these results confirm the presence of axonal transport defects in mice expressing FTD-linked mutant tau and demonstrate that traffic disruption is ameliorated by chronic administration of a specific p38α inhibitor, strengthening the link between this kinase and axonal transport regulation.

### Inhibition of p38α/p38β rescues axonal transport

**Fig. 5:**
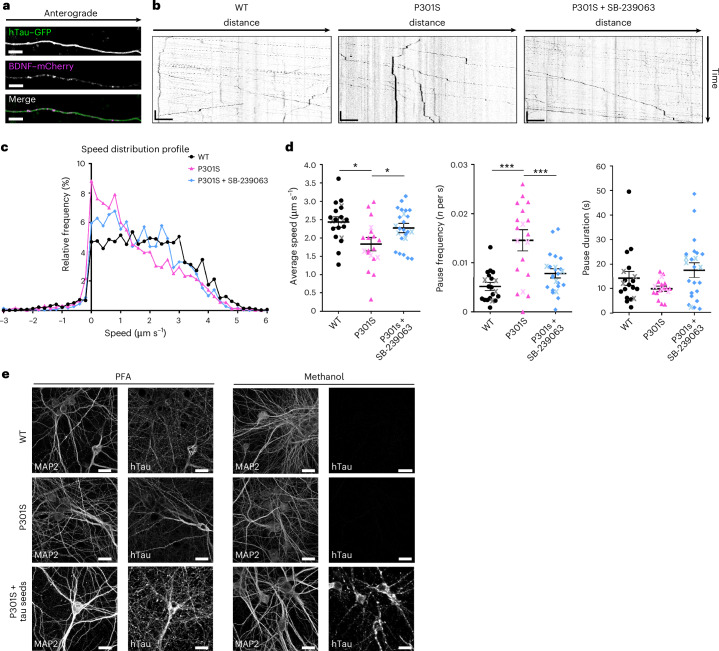
Inhibition of p38α/p38β rescues axonal transport in neuronal cultures. **a**, Confocal live imaging of a single axon of a days in vitro 21 (DIV21) mouse cortical neuron transfected at DIV7 with WT 1N4R hTau tagged with GFP (green) and BDNF–mCherry (magenta); scale bars, 5 µm. **b**, Kymographs of the axonal trafficking of BDNF–mCherry granules obtained by live imaging of axons as in **a**. Neurons were transfected at DIV7 with GFP-tagged human 1N4R tau, either WT or carrying the P301S mutation, and BDNF–mCherry. Neurons were treated for 2 h with either DMSO or 2 µM SB-239063 and subsequently imaged for 10 min at DIV21; scale bars, 10 µm (horizontal) and 20 s (vertical). **c**, Frequency distribution of the instantaneous speed of BDNF–mCherry-containing granule axonal transport; *n* = 16 neurons for WT, 15 neurons for P301S and 19 neurons for P301S + SB-239063 from four to five independent preparations per condition. **d**, Quantification relative to the data in **a**–**c**. Anterograde tracks were analyzed to calculate the values of the average speed (one-way ANOVA *F* = 4.037, *P* = 0.0241 Newman–Keuls multiple comparisons test), frequency of pausing (one-way ANOVA *F* = 11.85, *P* < 0.0001, Newman–Keuls multiple comparisons test) and the duration of pauses (Kruskal–Wallis test *P* = 0.3473); *n* = 16 neurons for WT, 15 neurons for P301S and 19 neurons for P301S + SB-239063 from four to five independent preparations per condition. Lighter ‘X’ symbols represent the averages per preparation. **e**, Confocal images of DIV21 mouse cortical neurons transduced at DIV7 with lentiviral particles encoding Flag-tagged human 1N4R tau, either WT or carrying the P301S mutation. The latter were either exposed or not exposed to tau seeds, corresponding to the sarkosyl-insoluble fraction of brain lysates obtained from rTg4510 mice. Neurons were fixed with either paraformaldehyde (PFA) or methanol, which extracts soluble proteins, leaving only aggregated tau to be detected, and then stained for MAP2 and Flag marking hTau; *n* = 3 independent preparations; scale bars, 20 µm. Bars represent the mean ± s.e.m. In **d**, the asterisks (*) indicate statistical significance as determined by one-way ANOVA with a post hoc correction for multiple comparisons (Newman–Keuls): *P < 0.05 and ***P < 0.001. Source data

**Extended Data Fig. 4:**
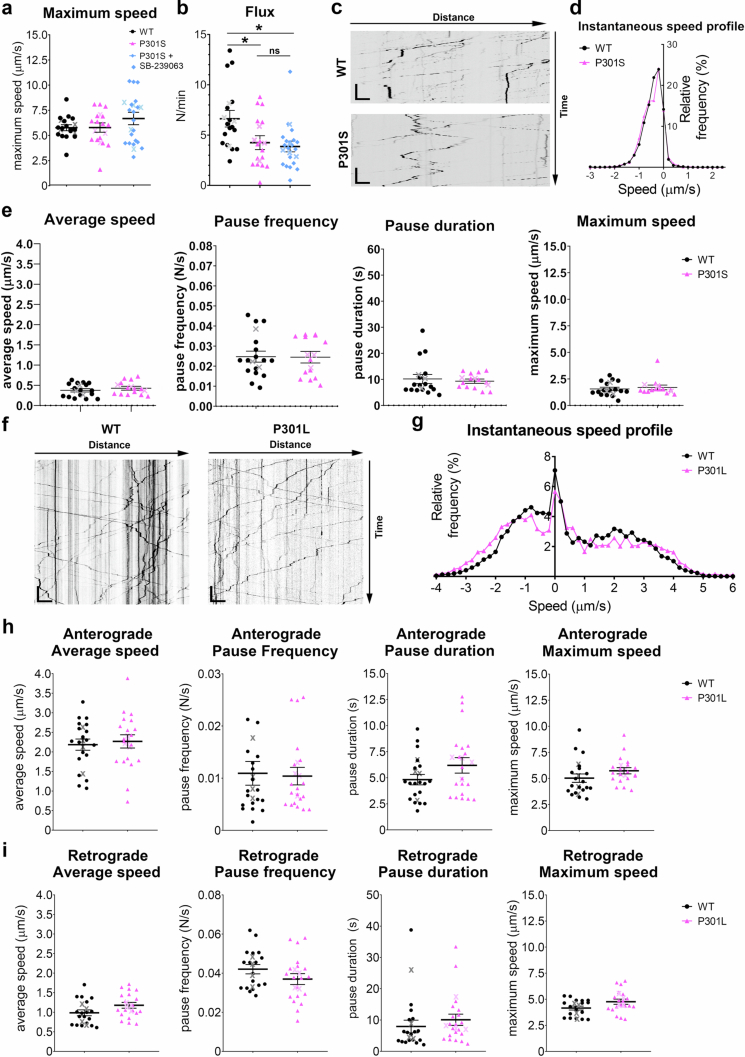
Inhibition of p38α/β rescues axonal transport in neuronal cultures. Related to Fig. 5. **a)** Quantification of the maximum speed of transport of BDNF-mCherry granules relative to Fig. 5**a-d**. (One-way ANOVA F = 1.159, p value = 0.3225). n = 16 neurons for WT, 15 neurons for P301S, 19 neurons for P301S + SB-239063 from 4-5 independent preparations per condition. **b)** Quantification of the flux (number of tracks per minute) of axonal axonal transport of BDNF-mCherry granules relative to Fig. 5**a-d**. (One-way ANOVA F = 4.851, p value = 0.0122, Newman-Keuls multiple comparison). n = 16 neurons for WT, 15 neurons for P301S, 19 neurons for P301S + SB-239063 from 4-5 independent preparations per condition. **c)** Kymographs of the retrograde axonal trafficking of BDNF-mCherry granules obtained by live imaging of axons similarly to Fig. 5a but with continuous bleaching of the most proximal part of the axon. Neurons were transfected at DIV7 with GFP-tagged human 1N4R tau either WT or carrying the P301S mutation, and BDNF-mCherry. Neurons were imaged for 10 min at DIV21. Scale bars = 4 µm (horizontal); 1 min (vertical). **d)** Frequency distribution of the instantaneous speed of BDNF-mCherry retrograde transport. **e)** Quantification relative to panel **d** of retrogradely moving organelles. Tracks were analysed to extract the values of the average speed (µm/s) (Unpaired two-tailed Student’s t-test p value = 0.4308), the frequency of pausing (N/s) (Unpaired two-tailed Student’s t-test p value = 0.9472), the duration of pauses (s) (Unpaired two-tailed Mann Whitney test p value = 0.442) and the maximum speed (µm/s) (Unpaired two-tailed Mann Whitney test p value = 0.4293). n = 16 and 13 neurons for WT and P301S, respectively from 3 independent preparations per group. Lighter X symbols represent the averages per preparation. **f)** Kymographs of LAMP1-mScarlet organelles obtained by live imaging of axons of DIV21 mouse cortical neurons transfected at DIV7 with human 1N4R tau either WT or carrying the P301L mutation and with LAMP1-mScarlet. Neurons were imaged for 10 min at DIV21. Scale bar = 10 µm (horizontal); 20 s (vertical). **g)** Frequency distribution of the instantaneous speed of LAMP1-mScarlet organelles transport. **h)** Quantification relative to panel **f-g** of anterogradely moving organelles. Tracks were analysed to extract the values of the average speed (µm/s) (Unpaired two-tailed Student’s t-test p value = 0.7126), the frequency of pausing (N/s) (Unpaired two-tailed Mann Whitney test p value = 0.8455), the duration of pauses (s) (Unpaired two-tailed Mann Whitney test p value = 0.297) and the maximum speed (µm/s) (Unpaired two-tailed Student’s t-test p value = 0.1683). n = 19 neurons from 4 independent preparations per group. Lighter X symbols represent the averages per preparation. **i)** Quantification relative to panel **f-g** of retrogradely moving organelles. Tracks were analysed to extract the values of the average speed (µm/s) (Unpaired two-tailed Student’s t-test p value = 0.0785), the frequency of pausing (N/s) (Unpaired two-tailed Student’s t-test p value = 0.1785), the duration of pauses (s) (Unpaired two-tailed Mann Whitney test p value = 0.1886) and the maximum speed (µm/s) (Unpaired two-tailed Student’s t-test p value = 0.0587). n = 19 neurons from 4 independent preparations per group. Lighter X symbols represent the averages per preparation. Bars represent the mean ± SEM. * = p < 0.05: statistical significance in one-way ANOVA with Newman-Keuls post-hoc correction for multiple comparison. Source data

To investigate the mechanistic basis of the axonal transport defects induced by mutant tau, we used mature mouse cortical neurons in culture in which we expressed GFP-tagged human WT tau or its FTD-associated P301S mutant and BDNF–mCherry, restricting our analysis to anterogradely moving organelles. As shown in Fig. 5a–d, mutant tau induced a reduction of the average speed of BDNF-containing secretory granules, an effect associated with an increase in pausing and a general reduction in the number of moving organelles (Fig. 5b–d and Extended Data Fig. 4a,b), similar to what we observed in vivo (Fig. 2) and to previous reports^54^. The speed and pausing defects were rescued by p38α/p38β inhibition, supporting that axonal transport is modulated by tau phosphorylation both in vitro and in vivo, and also in the context of P301S mutation.

To assess whether mutant tau could affect other organelles, we monitored the axonal retrograde transport of BDNF–mCherry^+^ vesicles, finding no differences between neurons expressing WT hTau or hTau-P301S (Extended Data Fig. 4c–e). Similarly, the transport of LAMP1-mScarlet^+^ compartments was also unaffected by the expression of mutant tau in this model system (Extended Data Fig. 4f–i). Thus, defects in axonal transport induced by mutant tau in this model system are not generalized to all organelles.

Additionally, we observed that the expression of WT or P301S tau was not sufficient to induce overt tau aggregation in mouse cortical neurons, as it was instead obtained by applying rTg4510-derived tau aggregates (Fig. 5e). This places these in vitro experiments within a pathological window similar to that when we first observed axonal transport defects in vivo (Fig. 2).

These data show that the impairment of the axonal transport of BDNF-containing secretory granules in primary cortical neurons extends to other tau mutants and that these impairments are rescued by p38 inhibition, as observed in vivo.

### Inhibition of p38α/p38β enhances axonal transport by decreasing mutant tau phosphorylation

**Fig. 6:**
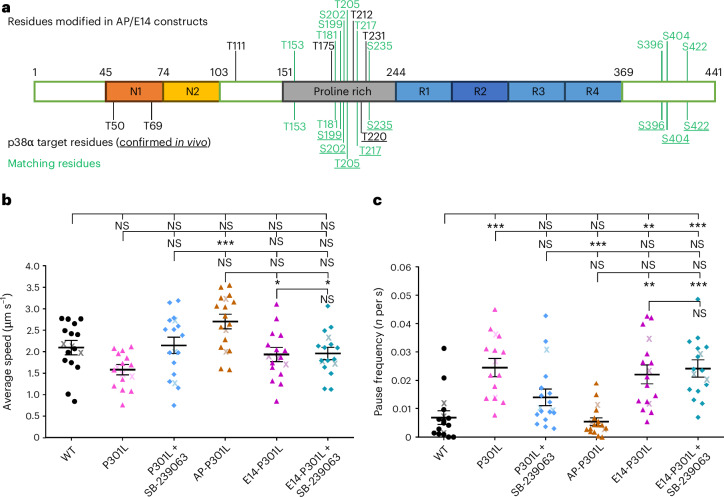
Inhibition of p38α/p38β enhances axonal transport by acting directly on tau phosphorylation. **a**, Schematic of the tau protein (2N4R isoform) structure with the main functional domains N1 and N2 (N-terminal repeats), proline-rich domain and R1–R4 (microtubule binding repeats) indicated. Above the schematic are shown residues modified in the AP (changed to alanine) or E14 (changed to glutamic acid) mutants. Below the schematic are indicated residues known to be phosphorylated by p38α from in vitro experiments. Residues in green are in common between the two groups. Ten of these 14 residues are known to be phosphorylated by p38; these are also 10 of the 13 residues known to be phosphorylated by p38 on tau. Underlined residues have been confirmed as p38α targets in vivo. **b**,**c**, Quantification of the axonal transport of BDNF–mCherry secretory granules in DIV21 mouse cortical neurons transfected at DIV7 with BDNF–mCherry and with 0N4R hTau tagged with GFP (WT, carrying the P301L mutation and/or with the AP or E14 mutation) and treated for 2 h with either DMSO or 2 µM SB-239063. Anterograde tracks were analyzed to extract the values of the average speed (**b**; one-way ANOVA *F* = 5.098, *P* = 0.0004 Holm–Sidak multiple comparisons test; adjusted *P* values: WT versus P301L *P* = 0.2753; WT versus P301L + SB-239063 *P* = 0.9665; WT versus AP-P301L *P* = 0.1092; WT versus E14-P301L *P* = 0.9309; WT versus E14-P301L + SB-239063 *P* = 0.9309; P301L versus P301L + SB-239063 *P* = 0.1611; P301L versus AP-P301L *P* = 0.0001; P301L versus E14-P301L *P* = 0.6515; P301L versus E14-P301L + SB-239063 *P* = 0.6220; P301L + SB-239063 versus AP-P301L *P* = 0.1611; P301L + SB-239063 versus E14-P301L *P* = 0.9240; P301L + SB-239063 versus E14-P301L + SB-239063 *P* = 0.9266; AP-P301L versus E14-P301L *P* = 0.0165; AP-P301L versus E14-P301L + SB-239063 *P* = 0.0214; E14-P301L versus E14-P301L + SB-239063 *P* = 0.9665) and the frequency of pausing (**c**; Kruskal–Wallis *P* < 0.0001 Dunn’s multiple comparisons test; adjusted *P* values: WT versus P301L *P* = 0.0009; WT versus P301L + SB-239063 *P* = 0.6618; WT versus AP-P301L *P* > 0.9999; WT versus E14-P301L *P* = 0.0056; WT versus E14-P301L + SB-239063 *P* = 0.0007; P301L versus P301L + SB-239063 *P* = 0.5332; P301L versus AP-P301L *P* = 0.0002; P301L versus E14-P301L *P* > 0.9999; P301L versus E14-P301L + SB-239063 *P* > 0.9999; P301L + SB-239063 versus AP-P301L *P* = 0.3307; P301L + SB-239063 versus E14-P301L *P* > 0.9999; P301L + SB-239063 versus E14-P301L + SB-239063 *P* = 0.4972; AP-P301L versus E14-P301L *P* = 0.0017; AP-P301L versus E14-P301L + SB-239063 *P* = 0.0002; E14-P301L versus E14-P301L + SB-239063 *P* > 0.9999); *n* = 14 for WT neurons, 13 for P301L neurons, 15 for P301L + SB-239063 neurons, 15 for AP-P301L neurons, 14 for E14-P301L neurons and 14 for E14-P301L + SB-239063 neurons from three independent preparations per condition. Lighter X symbols represent the averages per preparation. Bars represent the mean ± s.e.m. The asterisks (*) indicate statistical significance as assessed by one-way ANOVA with a post hoc correction for multiple comparisons: *P < 0.05, **P < 0.01 and ***P < 0.001; NS, not significant. Source data

To further investigate the link between tau phosphorylation, axonal transport and p38 activity, we used established tau mutants in which 14 serine and threonine residues, frequently hyperphosphorylated in AD, are mutated to either alanine (AP; phosphodeficient) or glutamic acid (E14; phosphomimetic)^50^. p38α has been shown to phosphorylate 10 of 14 of these residues^17^ (Fig. 6a)^17,18^.

**Extended Data Fig. 5:**
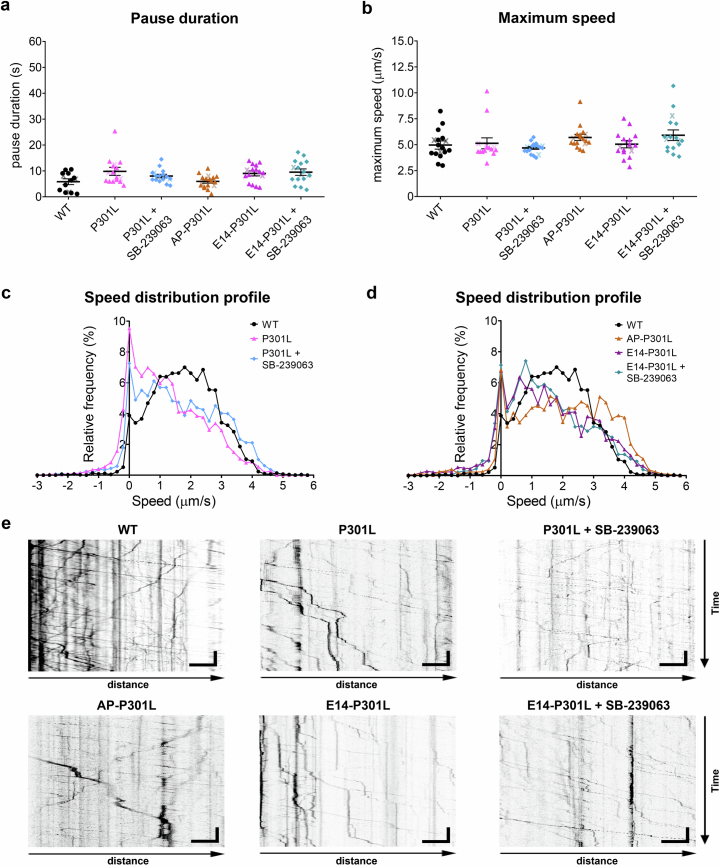
Inhibition of p38α/β enhances axonal transport by acting directly on tau phosphorylation. Related to Fig. 6. **a)** Quantification of the duration of pauses (s) (One-way ANOVA F = 2.688 p value = 0.0273, Holm Sidak multiple comparison adj p values: WT vs P301L = 0.1881, WT vs P301L + SB-239063 = 0.7692, WT vs AP-P301L = 0.9813, WT vs E14-P301L = 0.3784, WT vs E14-P301L + SB-239063 = 0.2315, P301L vs P301L + SB-239063 = 0.8325, P301L vs AP-P301L = 0.1469, P301L vs E14-P301L = 0.9728, P301L vs E14-P301L + SB-239063 = 0.9813, P301L + SB-239063 vs AP-P301L = 0.7692, P301L + SB-239063 vs E14-P301L = 0.966, P301L + SB-239063 vs E14-P301L + SB-239063 = 0.8851, AP-P301L vs E14-P301L = 0.3421, AP-P301L vs E14-P301L + SB-239063 = 0.1886, E14-P301L vs E14-P301L + SB-239063 = 0.9813) and **b)** the maximum speed (µm/s) (Kruskal Wallis test p value = 0.0488, Dunn’s multiple comparison adj p values: WT vs P301L > 0.9999; WT vs P301L + SB-239063 > 0.9999; WT vs AP-P301L = 0.4093; WT vs E14-P301L > 0.9999; WT vs E14-P301L + SB-239063 > 0.9999; P301L vs P301L + SB-239063 > 0.9999; P301L vs AP-P301L = 0.2143; P301L vs E14-P301L > 0.9999; P301L vs E14-P301L + SB-239063 = 0.8543; P301L + SB-239063 vs AP-P301L = 0.1920; P301L + SB-239063 vs E14-P301L > 0.9999; P301L + SB-239063 vs E14-P301L + SB-239063 = 0.8213; AP-P301L vs E14-P301L > 0.9999; AP-P301L vs E14-P301L + SB-239063 > 0.9999; E14-P301L vs E14-P301L + SB-239063 > 0.9999) relative to the experiment in Fig. 6. n = 14 for WT neurons, 13 for P301L neurons, 15 for P301L + SB-239063 neurons, 15 for AP-P301L neurons, 14 for E14-P301L neurons, 14 for E14-P301L + SB-239063 neurons from 3 independent preparations per condition. Lighter X symbols represent the averages per preparation. **c)** Frequency distribution of the instantaneous speed of BDNF-mCherry granules axonal transport relative to Fig. 6, panels **b-c** for WT, P301L and P301L + SB-239063 conditions. **d)** Frequency distribution of the instantaneous speed of BDNF-mCherry granules axonal transport relative to Fig. 6b-c for WT, AP-P301L, E14-P301L and E14-P301L + SB-239063 conditions. **e)** Kymographs of the axonal trafficking of BDNF-mCherry granules obtained by live imaging of axons relative to Fig. 6. Scale bars = 10 µm (horizontal); 20 s (vertical). Bars represent the mean ± SEM. Source data

We expressed BDNF–mCherry and GFP-tagged WT, P301L, AP-P301L or E14-P301L tau in primary mouse cortical neurons and treated them with either DMSO or SB-239063 (Fig. 6b,c and Extended Data Fig. 5). Pausing of BDNF-containing secretory granules was increased by the FTD-linked P301L mutant, similar to tau-P301S (Fig. 5a–d), an effect that was rescued by p38α/p38β inhibition, although it did not reach statistical significance. The phosphodeficient mutant (AP-P301L) abolished the effect of the P301L mutation, whereas the expression of E14-P301L tau caused impairments similar to the P301L mutation alone (Fig. 6b,c and Extended Data Fig. 5), as previously observed^6^. This effect was insensitive to p38α/p38β inhibition, demonstrating that the activity of SB-239063 on axonal transport is largely dependent on the reduction of P301L tau phosphorylation levels.

### Clusters of mutant tau on microtubules hinder axonal transport

**Fig. 7:**
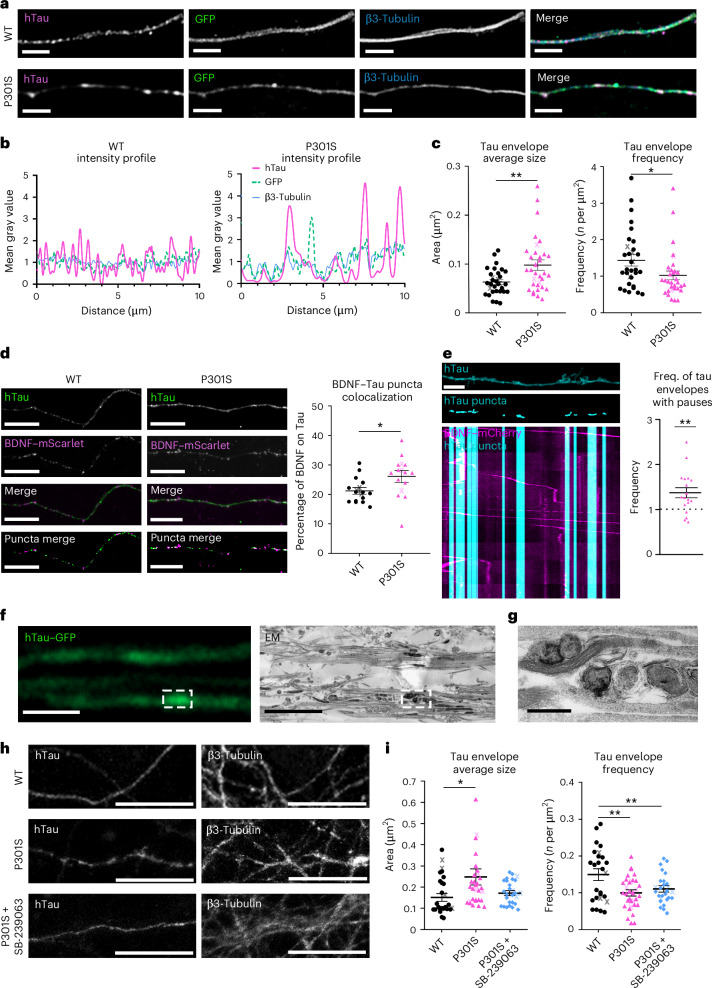
Mutant tau envelopes on microtubules act as obstacles for axonal transport. **a**, Airyscan images of axons of DIV15 mouse cortical neurons transduced at DIV7 with cytosolic GFP (green) and Flag-tagged human 1N4R tau (either WT or carrying the P301S mutation) and stained for Flag (hTau, magenta) and β3-tubulin (cyan). Mutant tau forms large clusters, which are referred to here as tau envelopes; scale bars, 2 µm. **b**, Signal intensities of hTau, GFP and β3-tubulin (each normalized to the mean intensity of the whole axon) along the length of the axons shown in **a**. **c**, Quantification of the average size (unpaired two-tailed Student’s *t*-test *P* = 0.0053) and frequency (unpaired two-tailed Mann–Whitney test *P* = 0.0258) of tau envelopes; *n* = 28 axons for WT and 30 axons for P301S from four independent preparations per group. Lighter X symbols represent the average per preparation. **d**, Left, confocal images of axons of DIV21 mouse cortical neurons transfected at DIV7 with BDNF–mScarlet (magenta) and Flag-tagged human 1N4R tau (either WT or carrying the P301S mutation) and stained for Flag (hTau, green). ‘Puncta merge’ shows the binarized signal used to evaluate the colocalization between tau envelopes (green) and BDNF–mScarlet granules (magenta); scale bars, 10 µm. Right, quantification relative to **d** of the object-based colocalization of BDNF–mScarlet granules on tau envelopes (percent; unpaired two-tailed Student’s *t*-test *P* = 0.0433); *n* = 14 axons for WT and 13 axons for P301S from three independent preparations per group. Lighter X symbols represent the average per preparation. **e**, Left, DIV7 mouse cortical neurons were transfected with Flag-tagged 1N4R tau carrying the P301S mutation and with BDNF–mCherry. At DIV21, anterograde axonal transport of BDNF–mCherry granules was imaged for 10 min using Airyscan, and kymographs were generated (tau is in magenta). Neurons were then fixed and stained with anti-Flag (cyan). Tau envelopes with a diameter larger than 0.36 µm were identified and binarized (hTau punta), and their position was correlated with the kymographs; scale bar, 5 µm. Right, quantification of the frequency of tau envelopes with pauses calculated as the ratio between the number of tau envelopes with pauses and the number of random axon sections of the same size with pauses. The dotted line represents the value expected (1) if the likelihood of tau envelopes to have a pause was the same as any other random section of the axon of similar size (one-sample *t*-test *P* = 0.0036); *n* = 18 neurons from five independent preparations. **f**, Correlative light and electron microscopy (EM) of axons of DIV15 mouse cortical neurons transduced at DIV7 with lentiviral particles encoding GFP-tagged human 1N4R tau carrying the P301S mutation. The experiment was repeated on three independent neuronal preparations; scale bars, 5 µm. **g**, Zoomed in electron micrograph of the region highlighted by the white rectangle in **f**; scale bar, 500 nm. **h**, Confocal images of axons of DIV21 mouse cortical neurons transfected at DIV7 with Flag-tagged human 1N4R tau (either WT or carrying the P301S mutation). Neurons were treated for 2 h with either DMSO or 2 µM SB-239063 and stained for Flag (hTau) and β3-tubulin; scale bars, 10 µm. **i**, Quantification of the average size (Kruskal–Wallis test *P* = 0.0286, Dunn’s multiple comparisons test adjusted *P* values: WT versus P301S *P* = 0.0241; WT versus P301S + SB-239063 *P* = 0.6695; P301S versus P301S + SB-239063 *P* = 0.4387) and frequency (one-way ANOVA *F* = 6.767, *P* = 0.0021, Newman–Keuls multiple comparisons test) of tau envelopes; *n* = 20 axons for WT, 26 axons for P301S and 23 axons for P301S + SB-239063 from four to five independent preparations per group. Lighter X symbols represent the average per preparation. Bars represent the mean ± s.e.m. In **c** and **d**, asterisks (*) indicate statistical significance in unpaired two-sided tests. In **e**, asterisks (*) indicate statistical significance in one-sample *t-*tests. In **i**, asterisks (*) indicate statistical significance as determined by one-way ANOVA or Kruskal–Wallis test followed with a post hoc correction for multiple comparisons; *P < 0.05 and **P < 0.01. Source data

We noticed that expression of both WT hTau and hTau-P301S in primary cortical neurons induced the formation of regions with high tau intensity along axons (Fig. 7a). This was surprising as tau is commonly reported to display a homogenous cytoplasmic signal along the axon. These regions did not overlap with areas of higher intensity of β3-tubulin or cytosolic GFP (Fig. 7b), ruling out axonal varicosities or plasma membrane blebs.

**Extended Data Fig. 6:**
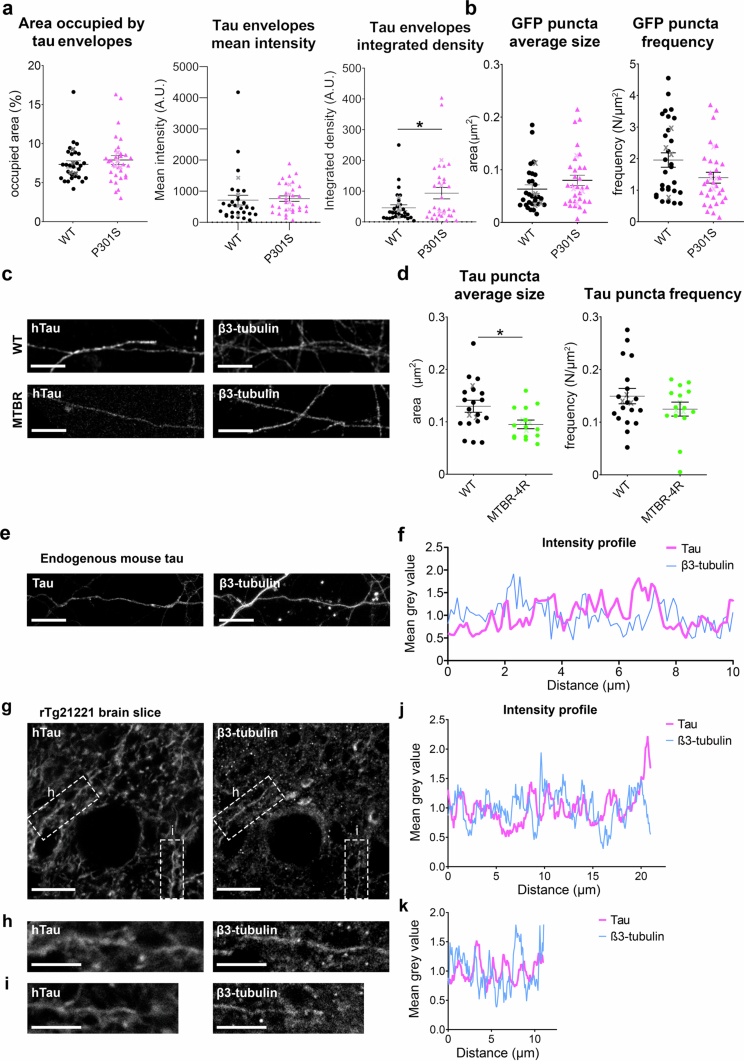
Mutant tau envelopes on microtubules act as obstacles for axonal transport. Related to Fig. 7. **a)** Quantification of the area of an axon segment occupied by tau envelopes (%) (Unpaired two-tailed Mann Whitney test p value = 0.444) and of the mean intensity (Unpaired two-tailed Mann Whitney test p value = 0.1511) and integrated intensity (Unpaired two-tailed Mann Whitney test p value = 0.0467) of the FLAG signal per tau envelope relative to Fig. 7a. n = 28 axons for WT, 30 axons for P301S from 4 independent preparations per group. **b)** Quantification of the average size (µm^2^) (Unpaired two-tailed Mann Whitney test p value = 0.2253) and frequency (N/µm^2^) (Unpaired two-tailed Mann Whitney test p value = 0.08) of GFP puncta relative to Fig. 7a. n = 28 axons for WT, 30 axons for P301S from 4 independent preparations per group. **c)** Confocal images of axons of DIV21 mouse cortical neurons transfected at DIV7 with either human 1N4R tau wild-type (WT) or with the 4 R microtubule binding region (MTBR-4R). Both proteins are FLAG tagged. Samples were then stained for FLAG (hTau) and β3-tubulin. Scale bar = 10 µm. **d)** Quantification relative to panel **c** of the average size (µm^2^) (Unpaired two-tailed Student’s t-test p value = 0.0277) and frequency (N/µm^2^) (Unpaired two-tailed Student’s t-test p value = 0.2336) of tau puncta. n = 18 axons for WT, 14 axons for MTBR from 3 independent preparations per group. Lighter X symbols represent the average per preparation. **e)** Confocal image of an axon of a DIV21 mouse cortical neuron stained for endogenous mouse tau and β3-tubulin. n = 4 mice. Scale bar = 10 µm. **f)** Signal intensities of mouse tau and β3-tubulin (each normalised to the mean intensity of the whole axon) along the length of the axon shown in panel **e**. **g)** Confocal image of a brain slice of a 4-month-old rTg21221 mouse transcardially perfused and stained for human tau and β3-tubulin. Scale bar = 10 µm. The insets in white depict the regions presented at higher magnification in panels **h** and **i**. **h)** Higher magnification of the axon from panel **g**. Scale bar = 5 µm. **i)** Higher magnification of the axon from panel **g**. Scale bar = 5 µm. **j)** Signal intensities of human tau and β3-tubulin (each normalised to the mean intensity of the whole axon) along the length of the axon showed in panel **h**. **k)** Signal intensities of human tau and β3-tubulin (each normalised to the mean intensity of the whole axon) along the length of the axon showed in panel **i**. Bars represent the mean ± SEM. In panel **a** and **d**, * indicate statistical significance in unpaired two-sided test. * = p < 0.05. Source data

Compared to WT tau, the P301S mutant forms larger tau puncta, although at a lower frequency along the axon (Fig. 7c), resulting in similar levels of axonal area coverage (Extended Data Fig. 6a). The intensity of tau signal per pixel in these puncta was not affected by the expression of mutant tau, suggesting equal density of tau inside these puncta, whereas the total intensity per puncta was increased due to the size increase (Extended Data Fig. 6a). The size and frequency of puncta of high GFP intensity were not affected by hTau-P301S, confirming the absence of morphological alterations (Extended Data Fig. 6b).

These tau^+^ structures were reminiscent of tau envelopes, recently described structures where several tau molecules bind cooperatively with high affinity to microtubules^28–30^. These structures assemble via tau–tau interactions not involving the microtubule binding region (MTBR). However, conclusive evidence demonstrating whether tau envelopes exist in neurons is lacking; furthermore, the impact of tau mutations, phosphorylation and aggregation on envelope formation is currently unclear.

To verify whether the observed structures were indeed tau envelopes, we expressed the MTBR domain of tau in mouse cortical neurons (Extended Data Fig. 6c). Regions characterized by high MTBR–tau intensity appeared significantly smaller than full-length tau (Extended Data Fig. 6c,d). In addition, we verified that tau envelopes are also formed by endogenous mouse tau, albeit less efficiently (Extended Data Fig. 6e,f). Similarly, heterogeneity in the tau signal along axons was detected in brain slices from rTg21221 mice that overexpress WT hTau (Extended Data Fig. 6g–k)^50^, suggesting that tau envelopes are present in nonpathological mouse brains.

**Extended Data Fig. 7:**
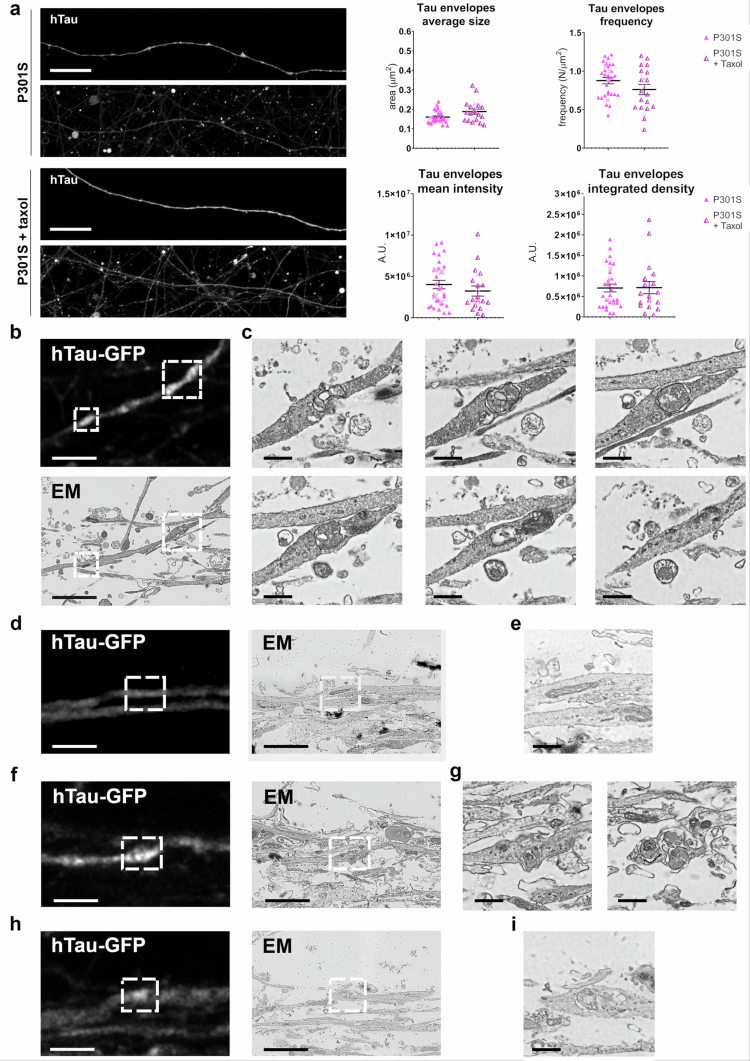
Mutant tau envelopes on microtubules act as obstacles for axonal transport. Related to Fig. 7. **a)** Left panel: Airyscan images of axons of DIV21 mouse cortical neurons transfected at DIV7 with FLAG-tagged human 1N4R tau carrying the P301S mutation and treated with either vehicle or 40 µM taxol for 4 h and stained for FLAG (hTau). Scale bar = 10 µm. Right panels: quantification of the average size (µm^2^) (Unpaired two-tailed Mann Whitney test p value = 0.0882), frequency (N/µm^2^) (Unpaired two-tailed Student’s t-test p value = 0.1284), mean intensity (A.U.) (Unpaired two-tailed Mann Whitney test p value = 0.2907) and Integrated density (A.U.) (Unpaired two-tailed Mann Whitney test p value = 0.7467) of tau envelopes. n = 28 axons for P301S, 18 axons for P301S + taxol from 3-4 independent preparations per group. Lighter X symbols represent the average per preparation. **b-i)** Additional examples of correlative light and electron microscopy on axons of DIV15 mouse cortical neurons transduced at DIV7 with lentiviral particles coding for GFP-tagged human 1N4R tau carrying the P301S mutation relative to Fig. 7f,g. Panel **c** shows a zoomed in electron micrograph of the region highlighted by the right white rectangle in panel **b**. Panels **e, g, i** show a zoomed in electron micrograph of the region highlighted by the white rectangle in panels **d, f, h**. Scale bar = 2.5 µm for panels **b, d, f, h**. Scale bar = 500 nm for panels **c, e, g, i**. Panels **c** and **g** show consecutive 70 nm thick Z sections. Bars represent the mean ± SEM. Source data

As tau envelopes were previously shown to be sensitive to the microtubule-stabilizing agent taxol^28,30^, we decided to test the effect of this compound on P301S tau envelopes (Extended Data Fig. 7a). We observed a trend toward increased size of tau envelopes and reduced frequency and mean intensity.

Previous work on tau envelopes showed that these structures affect axonal transport by competing with motor proteins for binding to microtubules^28–30^. We thus assessed whether the axonal transport defects induced by FTD-linked tau mutant P301S could be due to larger tau envelopes. Interestingly, BDNF granules and tau envelopes colocalized more when tau harbored the P301S mutation (Fig. 7d), suggesting that the observed increase in size of mutant tau envelopes drives the surge in pausing events and the reduction in axonal transport described in Fig. 5a–d.

To confirm this finding, we repeated the imaging of anterogradely moving BDNF–mCherry vesicles in neurons transfected with human P301S mutant tau. The videos were correlated with images acquired after hTau immunostaining to identify bona fide tau envelopes on the same axon. This analysis showed that areas occupied by tau-P301S envelopes are significantly more likely to display pausing events of BDNF–mCherry vesicles than any random region of the axon of the same size (mean probability 1.374 ± 0.111; Fig. 7e).

We then performed correlative light and electron microscopy of axons from neurons expressing GFP-tagged P301S tau (Fig. 7f,g). Regions of higher intensity of tau–GFP were frequently proximal to areas presenting accumulation of different organelles, supporting the hypothesis that tau envelopes function as obstacles for axonal transport also in neurons (Fig. 7f,g and Extended Data Fig. 7b–i).

Given the functional link between axonal transport impairments and tau envelopes, we decided to study the effect of p38 activity on these structures. We found that treatment with the p38α/p38β inhibitor SB-239063 caused the size of P301S mutant tau envelopes to trend toward WT tau levels with no effects on envelope frequency (Fig. 7h,i). These findings point toward a role of the P301S mutation in decreasing the rate of tau envelope formation, consistent with the reduced binding affinity of the tau-P301S mutant to microtubules^4^. The size of tau envelopes instead appears to be sensitive to p38 activity, and thus to tau phosphorylation status, in the presence of the P301S mutation.

Our results provide direct evidence for the presence of structures reminiscent of tau envelopes in primary neurons and in the adult mouse brain and demonstrate that tau envelopes function as obstacles for axonal transport. In addition, their size, which negatively correlates with the processivity of axonal transport, is increased by FTD-linked tau mutations and decreased by inhibiting p38-dependent mutant tau phosphorylation. Together, these findings further our mechanistic understanding of the axonal transport defects induced by pathological tau both in vitro and in vivo.

## Discussion

In this study, we showed that expression of FTD-linked tau-P301S/P301L mutants causes selective deficits in axonal transport in vivo at early stages of tau pathology preceding tau aggregation and neuronal activity alterations. These impairments are rescued by inhibition of p38α and consequent reduction of mutant tau phosphorylation. The latter is mechanistically linked to axonal transport deficits and the presence of larger tau envelopes in axons, which act as obstacles for axonal transport.

### P301S/P301L mutant tau envelopes affect axonal transport

We showed that tau is present in axons as discrete puncta (Fig. 7), which are reminiscent of tau envelopes^28–30^. These are regions where tau binds to microtubules with higher affinity and in a cooperative manner and are distinct from pathological aggregates^28–30^. We found that FTD-linked mutant tau-P301S formed sparser, yet larger, envelopes than WT tau (Fig. 7a–c). Similar structures were also observed in the mouse cortex (Extended Data Fig. 6g–k). Their morphology and the finding that tau MTBR assembled smaller puncta than full-length tau (Extended Data Fig. 6c,d) strongly suggest that these structures are tau envelopes. However, we cannot exclude that they may correspond to small pathological aggregates, potentially bound to microtubules. This could, for example, explain their response to taxol (Extended Data Fig. 7a), which is different from previous reports^30^. Although taxol was shown to disrupt WT tau envelopes in U2-OS cells^30^, the structures can assemble on taxol-stabilized microtubules^28–30^. Our experiment was performed with tau-P301S, which may alter the response of envelopes to taxol, explaining these differences.

Tau envelopes were frequently associated with organelle accumulation in their proximity (Fig. 7d–g and Extended Data Fig. 7b–i). This, together with our quantitative analysis of the axonal transport of BDNF–mCherry secretory granules (Figs. 5a–d and 7e), shows that larger tau envelopes are associated with anterograde axonal transport defects, in particular with an increase of pausing events. This effect is specific because the transport of other organelles was not affected by the expression of mutant tau (Extended Data Fig. 4c–i). Despite the absence of transport defects of LAMP1-mScarlet compartments, we cannot exclude impairments in lysosomal degradation, as previously observed in tauopathy models^55^. Interestingly, our correlative light and electron microscopy experiments (Fig. 7f,g and Extended Data Fig. 7b–i) showed accumulation of a range of different organelles immediately adjacent to tau envelopes, suggesting that the transport of other cargoes could be affected by larger tau envelopes. This finding likely reflects the diverse responsiveness of kinesin isoforms to tau envelopes^29^.

Tau envelope enlargement and axonal transport impairment are linked to tau mutation and phosphorylation, which may act in synergy. Consistently, inhibition of p38α/p38β restored the size of tau envelopes back to WT levels (Fig. 7h,i) and rescued axonal transport deficits (Fig. 5a–d). This effect is dependent on the reduction of mutant tau phosphorylation, as the axonal transport defects induced by a phosphomimetic mutant tau were insensitive to p38α/p38β inhibition (Fig. 6). Hence, this treatment could act on axonal transport via a direct action of p38α/p38β on tau or by other indirect mechanisms, such as the activity of other kinases downstream of p38α/p38β^20,56^ or via the masking of the tau phosphatase activating domain, which was previously shown to inhibit axonal transport^54,57^.

The correlation of axonal transport dynamics with the size of tau envelopes, rather than with their frequency (Fig. 7h,i), supports the existence of a threshold effect, whereby tau envelopes affect kinesin progression only when they reach a certain size. It is tempting to speculate that larger envelopes may constitute an early indicator of tau pathology and may facilitate aggregate formation. Recently published work using wild type tau in reconstituted microtubules and in human IPSCs-derived neurons confirms the presence of tau envelopes and the complex relationship between tau phosphorylation, envelope formation and axonal transport regulation^58–60^.

### In vivo axonal transport defects are caused by FTD-linked tau mutations

Despite several lines of in vitro evidence supporting a role for tau in regulating microtubule dynamics and axonal transport, in vivo experiments have been contradictory^8–11,61^. In this work, we used the AAV-driven expression of fluorescent BDNF in the mouse cortex and 2P imaging to increase spatiotemporal resolution (Fig. 1). Similar methods were recently used to study axonal transport of several organelles^62–65^. We chose to image transport in cortico-cortical projections for their relevance to tauopathies. However, this strategy limited our ability to establish transport directionality. Nevertheless, the transport speed observed (∼2.0 µm s^−1^; Figs. 2 and 3) was similar to that of anterograde neuropeptide Y-containing vesicles in vivo^64^ and of anterograde BDNF granules in cultured neurons (Fig. 5). By contrast, retrograde BDNF^+^ vesicles move at lower average speeds in cortical neurons (∼0.5 µm s^−1^; Extended Data Fig. 4c–e), strongly suggesting that most of the organelles tracked in our system are indeed moving anterogradely.

We first detected axonal transport deficits in 3-month-old rTg4510 mice (Fig. 2a–d). At this age, these mice present high levels of tau phosphorylation (Fig. 2e,f)^44,45^ but no overt tau aggregation (Fig. 2b), negligible neuronal loss and minor cognitive deficits^45,66^, thus recapitulating a very early disease stage. Crucially, we did not detect changes in neuronal activity (Extended Data Fig. 1a–c), as previously observed^49^. At this age, subtle neuronal activity deficits could only be detected using more sensitive electrophysiological approaches^67–69^.

Interestingly, axonal transport deficits are strictly dependent on mutant tau and did not seem to deteriorate over the course of tau pathology, with 5-month-old mice displaying deficits similar to younger animals (compare Figs. 3a–d and 2a–d). Five-month-old rTg4510 mice display overt neurofibrillary tangles in many, but not all, neurons^44,45^ (Fig. 3b). Therefore, neurons most affected may display a seemingly complete block of transport and therefore may not be accounted for in our analyses.

We have focused our attention on the axonal transport of BDNF, a neurotrophin crucial for neuronal homeostasis, synaptic plasticity and cognitive functions^37^. Deficits in BDNF axonal transport can result in its untimely and/or reduced secretion^37^, affecting the availability of survival signals and the ability of neuronal circuits to adapt, thus contributing to neurodegeneration. Future work could elucidate whether specific brain circuits show stronger axonal transport deficits than others.

Crucially, our work shows that axonal transport deficits are reversible both at early (Figs. 2 and 4) and more advanced stages of pathology (Fig. 3). We focused our attention on p38 inhibitors as this kinase is hyperactivated in neurons of individuals with tauopathy, where it associates with hyperphosphorylated tau^19–22^. Moreover, hyperactivated p38α negatively regulates axonal transport in an amyotrophic lateral sclerosis model^14^. This kinase phosphorylates tau both under physiological and pathological conditions^17,19–22^; however, its contribution to tauopathies was overshadowed by other kinases, such as GSK3β and CDK5 (refs. ^70–72^). Recently, the observation that p38α pharmacological or genetic inhibition was beneficial in AD and FTD models renewed attention of the field^24–27^. Interestingly, neuronal p38α can phosphorylate tau at several serine and threonine residues, promoting further phosphorylation^18^.

We used two different p38 inhibitors, including one approved for human use, in acute (Figs. 2 and 3) and long-term (Fig. 4) treatment protocols. Acute SB-239063 treatment was able to reduce p38α phosphorylation levels in both young and old rTg4510 mice in vivo (Figs. 2e,f and 3e,f and Extended Data Figs. 1e,f and 2e,f), likely by inhibiting its autophosphorylation^73^. Both compounds were effective in restoring axonal transport in vivo, although not completely (Figs. 2c,d and 3c,d and Extended Data Fig. 4). This partial effect suggests that other mechanisms, independent of tau phosphorylation, could contribute to axonal transport defects in these models. In addition, we limited the neflamapimod treatment to 5 days (Fig. 4), whereas others have extended the treatment to 4 weeks^23,48^. Nevertheless, our findings indicate that axonal transport restoration is one of the key mechanisms through which p38α inhibition exerts beneficial effects.

The early appearance of axonal transport deficits suggests that these impairments play a crucial role in the pathogenesis of tauopathies. As such, treatments reducing tau phosphorylation and restoring axonal transport could be particularly effective at early disease stages, potentially slowing down the rate of neuronal death and symptom progression. In this light, the link between axonal transport defects and mutant tau phosphorylation strongly supports the relevance of our results for other neurodegenerative diseases characterized by tau hyperphosphorylation, such as AD.

## Methods

### Experimental models

Experiments involving mice were performed under license from the UK Home Office in accordance with the Animals (Scientific Procedures) Act (1986) and were approved by the University College London (UCL) Queen Square Institute of Neurology Ethical Review Committee. Mice were maintained under a 12-h light/12-h dark cycle at constant room temperature (≈21 °C) with water and food provided ad libitum (Teklad Global 18% Protein Rodent Diet, Envigo, 2018C or Teklad Global 18% Protein Rodent Diet containing 200 mg per kg (body weight) doxycycline, TD.00502, Envigo). Cages were enriched with nesting material, plastic/cardboard tubes and wooden chew sticks as standard.

rTg4510 and rTg21221 mice were bred crossing C57BL/6 males carrying the CamKIIα-tet transgene in heterozygosity with FVB females carrying the hTau 0N4R P301L transgene or the hTau 0N4R WT transgene in heterozygosity, respectively. Genotypes were confirmed using real-time PCR with specific probes designed for each gene (Transnetyx).

Mouse primary cortical neurons were prepared from C57BL/6 embryos (embryonic day 16)^74^. Neurons were plated onto coverslips or in microfluidic devices (prepared as previously described^75^, coated overnight with 0.5 mg ml^−1^ poly-L-lysine; Sigma-Aldrich) at 75,000 per well or 100,000 per microfluidic device and grown in Neurobasal (Gibco) medium supplemented with 2% B27 (Gibco), 0.25% GlutaMAX (Gibco), 1% penicillin/streptomycin (Gibco) and 1% glucose (Sigma-Aldrich).

### Plasmids

pcDNA3-TauWT(1N4R)-Flag-GFP11, pcDNA3-TauP301S(1N4R)-Flag-GFP11, pHR- TauWT(1N4R)-Flag-GFP11 and pHR-TauP301S(1N4R)-Flag-GFP11 were previously described^75,76^. pHR-SFFV-GFP1-10 (Addgene plasmid 80409; http://n2t.net/addgene:80409; RRID: Addgene_80409) and pHR-hSyn-EGFP (Addgene plasmid 114215; http://n2t.net/addgene:114215; RRID: Addgene_114215) were kind gifts from B. Huang (Department of Pharmaceutical Chemistry, University of California San Francisco, San Francisco, CA, USA) and X. Han (Department of Biomedical Engineering, Boston University, Boston, MA, USA), respectively. pAAV-CamKIIα-BDNF-mCherry was assembled by PCR cloning from pJPA5-BDNF-mCherry (a gift of M. Verhage; Department of Functional Genomics, Center for Neurogenomics and Cognitive Research (CNCR), Vrije Universiteit (VU) Amsterdam, Amsterdam, the Netherlands) into pAAV-CaMKIIa-EGFP (a gift from B. Roth; Department of Pharmacology, School of Medicine, University of North Carolina at Chapel Hill, Chapel Hill, NC, USA; Addgene plasmid 50469; http://n2t.net/addgene:50469; RRID: Addgene_50469). pAAV-CamKIIα-BDNF-mScarletI was assembled from LAMP1-mScarlet (a gift from D. Gadella; Swammerdam Institute for Life Sciences, Section of Molecular Cytology, van Leeuwenhoek Centre for Advanced Microscopy, University of Amsterdam, Amsterdam, the Netherlands; Addgene plasmid 98827; http://n2t.net/addgene:98827; RRID: Addgene_98827) into pAAV-CamKIIα-BDNF-mCherry. pAAV-CamKIIα-Lamp1-mScarletI was assembled by inserting LAMP1-mScarletI into pAAV-CaMKIIa-EGFP. pcDNA3-MTBR-WT(1N4R)-Flag-GFP11 was assembled by inserting MTBR-4R into pcDNA3-TauWT(1N4R)-Flag-GFP11. pRK5-EGFP-Tau, pRK5-EGFP-Tau P301L, pRK5-EGFP-Tau AP-P301L and pRK5-EGFP-Tau E14-P301L were gifts from K. Ashe (Department of Neurology, University of Minnesota, Minneapolis, MN, USA) (Addgene plasmid 46904, http://n2t.net/addgene:46904, RRID: Addgene_46904; Addgene plasmid 46908, http://n2t.net/addgene:46908, RRID: Addgene_46908; Addgene plasmid 46906, http://n2t.net/addgene:46906, RRID: Addgene_46906; Addgene plasmid 46909, http://n2t.net/addgene:46909, RRID: Addgene_46909).

### Transfection and infection

For lentivirus production, Lenti-X 293T cells (Takara Bio) were grown in DMEM supplemented with 10% fetal bovine serum, 1% L-glutamine and 0.1% gentamycin (Invitrogen) and transfected using Lipofectamine 3000 (Thermo Fisher Scientific) following the manufacturer’s instructions. Viral particles were recovered using a Lenti-X Concentrator (Takara Bio) following the manufacturer’s instructions.

Mouse cortical neurons were transfected with Lipofectamine 2000 (Thermo Fisher Scientific). Briefly, for 1 well of a 12-well plate, 1.5–2 μg of DNA and 2 μl of Lipofectamine 2000 were mixed in 200 μl of Neurobasal (Gibco) and incubated for 15 min before being applied to DIV7 neurons for 30 min in transfection medium made of 80% Neurobasal and 20% conditioned medium.

### Tau seed preparation

Tau seeds were purified through a sarkosyl extraction protocol (adapted from ref. ^77^) from the forebrains of 21-week-old female rTg4510 mice^44^. First, forebrains were thawed via heat shock at 37 °C for 90 s. Brains were homogenized with a Teflon glass dounce homogenizer in RIPA buffer (50 mM Tris-HCl (pH 7.4), 150 mM NaCl, 1 mM EDTA, 1% NP-40, 1% Triton X-100 and protease and phosphatase inhibitors) with 100 mg of forebrain tissue used per ml of RIPA. Homogenates were centrifuged at 20,000*g* at 4 °C for 20 min. The supernatant was incubated in 1% sarkosyl on a rotator for 1 h at room temperature and then centrifuged at 100,000*g* at 4 °C for 90 min. Pellets were washed three times with PBS and resuspended in PBS. The suspended pellet was sonicated (Q800R3 Sonicator, Qsonica) for 5 min at 60% amplitude at 4 °C. This pellet was washed an additional three times before being resonicated (2 min, 60% amplitude, 4 °C), aliquoted, snap-frozen and stored at –80 °C. Total protein concentration was determined by a BCA quantification kit following the manufacturer’s instructions (Thermo Fisher Scientific). Sarkosyl-insoluble tau aggregates (10 μg) were further sonicated as described earlier before being applied to neurons at DIV 6.

### Tissue lysis

Neuronal cultures were washed in PBS before being scraped off in RIPA buffer supplemented with protease and phosphatase inhibitors. Lysates were incubated on a rotator for 30 min at 4 °C and centrifuged at 16,000*g* for 15 min to remove cell debris.

For analysis of mouse brains, animals were killed by cervical dislocation, and the forebrain was quickly removed, washed in PBS, collected in cryovials and snap-frozen in liquid nitrogen. Samples were stored at –70 °C until use. Snap-frozen forebrains were quickly thawed and transferred to Lysis Matrix D tubes (116913100, MP Biomedicals) with 1 ml of RIPA buffer supplemented with cOmplete Mini proteases inhibitors (11836170001, Roche) and phosphatases inhibitor cocktail (524625, Merck Millipore). Forebrains were lysed using a Precellys Tissue Homogenizer via three cycles of 30 s ON/120 s OFF at 6,000 rpm at 4 °C. After centrifugation, the supernatants were transferred to 15-ml tubes with 2 ml of fresh RIPA buffer, vortexed and incubated on ice for 30 min.

For 3-month-old forebrains, 1 ml of crude homogenate was centrifuged for 30 min at 21,000*g* at 4 °C, and the protein concentration in the supernatant was quantified via Pierce BCA Protein Assay. Ten micrograms of protein was mixed with Laemmli buffer (1610747, Bio-Rad) and 50 mM DTT (R0861, Thermo Scientific) and heated for 5 min at 95 °C. For 5-month-old forebrains, 6 µl of crude homogenate was mixed with Laemmli buffer with 50 mM DTT and heated for 5 min at 95 °C.

For sarkosyl-soluble and sarkosyl-insoluble analysis, 500 µl of crude homogenate was diluted up to 1 ml in RIPA buffer supplemented with protease and phosphatase inhibitors and 1% sarkosyl NL30 (442753R, BDH) in ultracentrifuge tubes (357448, Beckman Coulter). Samples were incubated for 1 h with rotation at room temperature, followed by ultracentrifugation using an Optima MAX-XP benchtop ultracentrifuge (Beckman Coulter) with a TLA-55 rotor for 90 min at 150,000*g* and 4 °C. Proteins in the sarkosyl-soluble supernatants were collected, and 1/75 of the volume was mixed with Laemmli buffer with DTT and heated for 5 min at 95 °C. The sarkosyl-insoluble pellets were resuspended in 200 µl of Laemmli buffer with DTT, heated for 10 min at 95 °C and sonicated using a QSonica water bath sonicator (five cycles of 30 s ON/30 s OFF at 80% power) at 4 °C. Once the pellet was completely dissolved, 1/75 of the volume was diluted with Laemmli buffer and DTT.

### Western blotting

Proteins were transferred from SDS–PAGE acrylamide gels (Bio-Rad) onto nitrocellulose membranes (0.22 µm; GE Healthcare) or PVDF membranes. Membranes were then incubated with primary antibodies (anti-p38α, Cell Signaling, 9218, 1:1,000; anti-p-p38, Cell Signaling, 4511, 1:500; anti-GAPDH, Abcam, ab245355, 1:1,000; anti-hTau, Biolegend, 15-25 Antibody TAU13, 1:500; anti-AT8, Thermo Fisher Scientific, 1:500; anti-total tau DA9, 1:10,000, a kind gift from Eli Lilly) at room temperature for 2 h or overnight at 4 °C in TBS supplemented with 0.1% Tween-20 (Sigma-Aldrich) and 5% bovine serum albumin (BSA; Sigma-Aldrich). After washing, the blots were incubated at room temperature for 1 h with horseradish peroxidase-conjugated anti-rabbit or anti-mouse (1:2,000/1:5,000) in TBS supplemented with 0.1% Tween-20 and 5% BSA. For Coomassie staining, membranes were incubated for 1 min with 1% Coomassie Brilliant Blue (R-250, Bio-Rad) in 7% acetic acid and 40% methanol, followed by destaining with 7% acetic acid and 40% methanol. The immunoreactive bands on blots were visualized by enhanced chemiluminescence (Luminata Crescendo, Merck Millipore) using an Amersham Imager 600 (GE Healthcare). The intensity of the bands was measured with ImageJ using the ‘Analyze – Gels’ tools.

### Immunocytochemistry

Cultured neurons were washed in PBS and fixed in 4% PFA (Sigma-Aldrich)/10% sucrose (Sigma-Aldrich) for 10 min at room temperature or with 100% methanol at –20 °C to extract soluble proteins and leave only aggregated tau to be detected^77^. After three washes in PBS, cells were permeabilized in PBS, 0.5% BSA, 0.2% Triton X-100 and 10% goat serum (Gibco) or in 5% BSA and 0.1% saponin in PBS and incubated with primary antibodies (anti-Flag, M2 F1804, Sigma-Aldrich, 1:1,000; anti-β3-tubulin, Biolegend, clone TU27/tubulin, 1:500; anti-tau, Dako, A0024, 1:5,000; anti-hTau, 15-25 antibody clone TAU13 Biolegend, 1:250) in PBS, 0.5% BSA and 10% goat serum for 2 h at room temperature or overnight at 4 °C. After three washes with PBS, coverslips were incubated with AlexaFluor-conjugated secondary antibodies (1:400) in PBS, 0.5% BSA and 10% goat serum for 1 h at room temperature. Coverslips were washed with PBS and mounted with Mowiol (Sigma-Aldrich).

Thirty-micron-thick coronal brain slices were obtained from cryostat (Leica) sectioning of brains from animals transcardially perfused with PBS and 4% PFA in PBS after cryoprotection in 30% sucrose. Slices were subsequently washed in PBS for 20 min, in 1% Triton X-100 in PBS for 20 min, in PBS for 5 min and in 0.3% Triton X-100 in PBS for 30 min and then blocked in 20% goat serum, 1% BSA and 0.3% Triton X-100 in PBS for 2 h. Primary antibodies (anti-hTau, 15-25 clone TAU13, Biolegend, 1:250; anti-β3-tubulin, Sigma-Aldrich, T2200, 1:1,000; anti-GFP, Aves GFP-1020, 1:1,000; anti-mScarlet, Synaptic Systems N1302-AF568-L, 1:250; anti-NeuN, Abcam, ab128886, 1:1,000; anti-Alz50, 1:7,500) were diluted in 20% goat serum, 1% BSA and 0.3% Triton X-100 in PBS and incubated with brain slices over the weekend at 4 °C.

Sections were then washed with PBS for 10 min and 0.3% Triton X-100 in PBS for 30 min before being incubated with AlexaFluor-conjugated secondary antibodies (1:500) diluted in 20% goat serum, 1% BSA and 0.3% Triton X-100 in PBS for 4 h at room temperature. Sections were then washed in PBS for 10 min and in 0.3% Triton X-100 in PBS for 15 min before being rinsed in water and mounted with Fluoromount G (Sigma-Aldrich).

Fluorescence images were acquired with an LSM880 microscope (Carl Zeiss) and a ×63/1.4-NA oil immersion objective either in confocal (Figs. 1b–d,j, 2b, 3b, 5a,e and 7f,h and Extended Data Figs. 1g and 6c,e,g–i) or Airyscan mode (Fig. 7a,e and Extended Data Fig. 7a,b,d,f,h) or with a Leica Stellaris 8 in confocal mode with a ×100 objective (Fig. 7d). Images were collected as *z*-stack series projections of approximately six to ten images taken at depth intervals of 0.75 μm.

Tau envelopes were identified as follows. The β3-tubulin signal was used to define a mask of the axon. Tau signal was then measured, and the images were binarized, with threshold = mean + three times the standard deviation when images were acquired by standard confocal microscopy or with threshold = mean + standard deviation for images acquired via Airyscan microscopy.

The intensity profiles presented in Fig. 7b and Extended Data Fig. 6f,j,k were generated in ImageJ by drawing a line across the length of an axon and using the ‘Analyze – Plot profile’ tool. The line was made of thickness sufficient to cover the whole axon.

Colocalization in Fig. 7d was calculated by binarizing the tau signal as described earlier and binarizing the BDNF–mScarlet signal using threshold = mean + standard deviation. Colocalization was then calculated using the ImageJ plugin Jacop^78^ with the ‘Center of mass’ and ‘Work on distances between centers’ options selected.

### Live imaging of cultured neurons

Neurons were plated in glass-bottomed dishes and transfected as described for each experiment. At DIV21, the medium was replaced by Tyrode’s buffer (150 mM D-glucose, 108 mM NaCl, 5 mM KCl, 2 mM MgCl_2_, 2 mM CaCl_2_ and 25 mM HEPES-NaOH, pH 7.4) and left to equilibrate at 5% CO_2_ and 37 °C. A 130-μm-long stretch of the axon of GFP^+^ and mCherry^+^ neurons was imaged with an LSM880 microscope in Airyscan fast modality with an acquisition rate of one image every 300/600 ms and 0.097-μm pixel size using a ×63 objective. For the imaging of BDNF–mCherry transport, the more distal portion of the axon was continuously bleached with a low-power laser to exclude retrogradely moving organelles. Transport was analyzed using the ImageJ plugin Kymoanalyser^79^, randomly selecting between 10 and 30 tracks per kymograph. Videos with less than ten tracks were excluded from the analysis. Pauses were defined as segments in which the velocity was less than 0.1 μm s^−1^. Each data point presented for average and maximum speed values corresponds to the average of all tracks from one individual axon. Similarly, each data point presented for pause frequency and pause duration is the average of all tracks followed in the same axon. Instantaneous speed profiles display the relative frequency of each bin of velocity (0.2 μm s^−1^) averaging all the tracks from one individual axon. For the analysis of flux, we used the plugin Kymobutler to extract all tracks from the generated kymographs and then automatically counted the number of tracks crossing a vertical line positioned at *x* = 15 pixels.

### Correlative light electron microscopy

Primary mouse cortical neurons were plated on microfluidic devices mounted over a photoetched coverslip with a grid of 200 numbered squares of 500 μm (Electron Microscopy Science), transduced with lentiviral particles encoding GFP-tagged 1N4R-P301S-tau at DIV7 and fixed at DIV15 in 4% electron microscopy-grade PFA/10% sucrose in PBS for 15 min. Fluorescence and DIC images were acquired, and regions of higher GFP intensity, corresponding to tau envelopes, were identified in relation to their position on the grid. Samples were processed for transmission electron microscopy (Fig. 7f,g) as detailed in ref.^80^ or for array tomography scanning electron microscopy (Extended Data Fig. 7b–i) as detailed in refs. ^81,82^. The superimposition of fluorescence and DIC images allowed relocation of the high-GFP-intensity areas when trimming blocks for sectioning and transmission electron microscopy imaging by using the grid marks transferred from the coverslips.

### Animal surgery and 2P imaging

Surgical and experimental procedures were conducted in accordance with the Animal (Scientific Procedures) Act (1986), approved by the UCL Animal Welfare and Ethical Review Body and performed under an approved UK Home Office project licence (GS). The serotype 1 AAV for axonal–GCaMP6s (Ax-GCaMP6 AAV) expression, under the control of a *SYN1* promoter, was obtained from Addgene^36^. The serotype 1 AAV for BDNF–mScarlet, under the control of a *CAMK2A* promoter, was produced in HEK293T cells by the UCL NeuroGTx Vector Core Facility, UCL School of Pharmacy, following standard procedures, and purified using an iodixanol gradient.

For surgeries, anesthesia was induced and maintained using gaseous isoflurane. Animals received a subcutaneous injection of meloxicam (Metacam, 2 mg ml^−1^; 5 mg per kg (body weight)), an intramuscular injection in the thigh of dexamethasone (Colvasone, 2 mg ml^−1^; 0.5 mg per kg (body weight)) and a subcutaneous injection of lidocaine (1%) just above the skull. The mouse was then placed in a stereotactic frame over a heating pad. The skin above the skull was incised, and the skull was exposed. The connective tissue was removed, and the area was cleaned with sterile ice-cold Dulbecco’s PBS (Gibco). The coordinates for the V2mm were marked on the skull at anterior–posterior = –3 and lateral = 1.3 from bregma. Using this point as the most posterior–medial point, a circular hole of around 4 mm in diameter was drilled in the skull, avoiding the coronal and sagittal sutures. This would ensure that the LPtA remained at the center of the craniotomy. The piece of skull was removed, and the area was immediately covered with a hemostatic sponge soaked in ice-cold Dulbecco’s PBS. Once any eventual bleeding had stopped, the syringe was loaded with premixed 0.5 μl of Ax-GCaMP6 AAV (8.5 × 10^11^ viral genomes per ml) and 0.5 μl of BDNF-mScarlet AAV (5.09 × 10^11^ viral genomes per ml), which was injected with an automatic pump at 200 nl min^−1^ at anterior–posterior = –3, lateral = 1.3 and dorsal = –0.75. After resting for an additional 5 min, the syringe was retracted, and a 5-mm round glass coverslip was positioned over the craniotomy and glued to the skull with cyanoacrylate glue (Vetbond). The margins of the skin were glued to the skull, and the exposed skull was covered with dental cement (Superbond, Prestige Dental), which also covered the edges of the glass coverslip to create a well. The anesthetic was then stopped, and the mouse was removed from the stereotactic frame. The animal received a subcutaneous injection of buprenorphine (Vetergesic, 3 mg ml^−1^; 0.1 mg per kg (body weight)) for post-operative pain relief and was placed back into a clean cage on a heated rack to be monitored until recovery. Mice received meloxicam (Metacam, 1.5 mg ml^−1^) orally for 3 days after surgery and were fed a wet diet to aid recovery. Twenty-one days after surgery, animals were anesthetized with gaseous isoflurane (4%) and placed on an heating pad with the head secured in a stereotaxic frame and ear bars. The coverslip was cleaned with 70% ethanol, and clear ultrasound gel was placed over it. The mouse was then placed under a custom-built resonant-scanning 2P microscope (Independent NeuroScience Services) controlled by ScanImage (MBF Bioscience) software and equipped with a Coherent Chameleon Discovery NX tunable laser and a ×16/0.8-NA Nikon water immersion objective. The objective was positioned at the center of the coverslip, and axonal–GCaMP6 and BDNF–mScarlet signals were assessed at 920 nm and 1,100 nm, respectively, at about 100 μm depth from the surface. The anesthesia was then slowly reduced (~0.25% every 20 min) until 0.8% isoflurane was reached. Three fields of view of 98.5 μm × 98.5 μm were imaged in the two channels separately (for 5 min at 920 nm and 10 min at 1,100 nm) at 512 × 512 pixels with a 30-Hz acquisition rate.

### Drug treatment

For experiments in cultured neurons, SB-239063 (GlaxoSmithKline) was dissolved in DMSO at 1 mM and used at a final concentration of 2 μM, taxol (1097, Tocris) was dissolved in DMSO at 50 mM and used at a final concentration of 40 μM. For in vivo experiments, SB-239063 was suspended at 0.1 mg ml^−1^ in 1% methylcellulose (Sigma-Aldrich), which was injected intraperitoneally 1.45–2 h before the experiment at a dose of 100 mg per kg (body weight). The dosage, route and timing of administration were selected based on previous pharmacokinetic studies^47^. Control and pretreatment animals were injected with the same volume of 1% methylcellulose. Neflamapimod (CervoMed) was resuspended at 0.6 mg ml^−1^ in 1% Pluronic F108 (Sigma-Aldrich) and administered at a concentration of 3 mg per kg (body weight) via oral gavage twice per day for 5 days. The experiments started around 2 h from the last dose. A group of CamKIIα-tet animals received 1% Pluronic F108 via oral gavage twice per day for 5 days, and the imaging started 2 h from the last dose.

### In vivo imaging analysis

Videos of axonal calcium transients were processed with Suite2P^83^, an image processing pipeline written in Python 3 specifically developed for calcium imaging analysis. Registration and identification of the ROIs was performed with the default settings specifying only the ‘tau’ value of 1.35 corresponding to the recommended timescale for GCaMP6. The extracted *F* values were then analyzed with a custom MATLAB script. Briefly, the median of the lowest 5% *F* values was used as *F*_0_, and the Δ*F*/*F*_0_ was calculated as (*F* – *F*_0_) / *F*_0_. Δ*F*/*F*_0_ peaks were considered events if they were above the median + 4 s.d. of the whole recording. Peaks were considered separate events if they were separated by more than 0.9 s. The frequency of calcium transients was then calculated as the total number of events divided by the recording time.

For videos of the BDNF–mScarlet granules, frames were first averaged in groups of 20, bringing the actual time frame rate to 0.6 s. Each movie of 10 min was divided into three segments of 3.33 min each, and for each segment, the ten most intense organelles were manually tracked using the ImageJ plugin Trackmate. Thirty organelles were tracked per field of view. As three fields of view were imaged for each animal, a total of 90 organelles were tracked per animal. A pause was defined if the frame-to-frame speed was lower than 0.4 μm s^−1^ for two consecutive segments. The average speed, maximum speed, frequency of pauses and duration of pauses of all tracks in one field of view were averaged. The values from the three fields of view of each animal were then averaged and plotted. The instantaneous speed profiles plot the relative frequency of each frame-by-frame velocity bin (interval of 0.4 μm s^−1^) among all the tracks averaged from one animal.

### Automated transport analysis pipeline

This workflow, which is based on the ImageJ plugin Trackmate, detects all moving objects according to set criteria, thus producing a value representative of the number of moving organelles per field of view in the recorded time frame. This value can be considered an indicator of the flux of organelles transported by axons at any given time.

Frames were first averaged in groups of 20, bringing the actual time frame rate to 0.6 s. The videos were then registered with Suite2p^83^ using default settings and filtered for variance between two consecutive frames using the ‘Variance 3D’ command on ImageJ. The resulting videos were inputted in Trackmate using the Stardist algorithm for segmentation and detection of objects using default settings. Objects were then included only if they had a radius of >0.1 μm and <1.6 μm. The Kalman tracker was used with ‘Max frame gap’ of five frames, ‘Kalman search radius’ of 3.5 μm and ‘Linking max distance’ of 1 μm. Tracks were then filtered for ‘track displacement’ > 6 μm and for ‘maximum speed’ < 6 μm s^−1^. After the videos were processed, we manually curated the tracks to remove those generated erroneously over very bright and large objects, likely to correspond to cell bodies. The number of tracks confirmed was divided by the duration of the video in minutes to generate the track frequency value that is presented in Extended Data Fig. 3.

### Statistical analyses

All statistical analyses were performed using GraphPad Prism 8. Normality of residuals was assessed using the Shapiro–Wilk test (for datasets with less than six values) or the Kolmogorov–Smirnov test, and the data met the assumptions of the statistical tests used. Comparisons between two groups following a normal distribution were analyzed using two-tailed unpaired *t*-tests, whereas comparisons between two groups not following a normal distribution were analyzed using a Mann–Whitney test. When three groups were compared and data were normally distributed, an ordinary one-way ANOVA was used; if significant, it was followed by a Newman–Keuls multiple comparisons test, with the exception of western blot data, which were analyzed by the two-stage linear step-up procedure of Benjamini, Krieger and Yekutieli multiple comparisons. When three groups were compared and data were not normally distributed, ranks were compared to the Kruskal–Wallis test, followed by a Dunn’s multiple comparisons test. When four or more groups were compared and the data followed a normal distribution, they were compared with one-way ANOVA followed by Holm–Sidak correction for multiple comparison. When four or more groups were compared and the data were not normally distributed, ranks were compared to the Kruskal–Wallis test, followed by a Dunn’s multiple comparisons test.

Paired Student’s *t*-tests (if data were normally distributed) or a Wilcoxon matched-pairs signed-rank test (if data were not normally distributed) was used to compare the animals before and after treatment in Figs. 2d, 3d and 4c,d and Extended Data Figs. 1d, 2d and 3d. A one-sample *t*-test was used to assess statistical significance between one group and a theoretical value of 1 in Fig. 7e. Numerosity of the samples is detailed in the legend of each figure. The specific tests and *P* values are listed in the figure legends.

All analyses were conducted blind to the experimental condition. When appropriate, animals were randomly assigned to conditions, and conditions were randomized to account for potential ordering effects. To avoid litter bias in the mouse experiments, experimental groups were composed of animals from different litters, which were randomly distributed. No statistical methods were used to predetermine sample sizes, but our sample sizes are similar to those reported in previous publications. In Fig. 2e,f, one sample was excluded using the Grubb’s test, as it was determined that the animal did not respond to the SB-239063 injection, likely due to an error in the injection.

### Reporting summary

Further information on research design is available in the Nature Portfolio Reporting Summary linked to this article.

## Online content

Any methods, additional references, Nature Portfolio reporting summaries, source data, extended data, supplementary information, acknowledgements, peer review information; details of author contributions and competing interests; and statements of data and code availability are available at 10.1038/s41593-026-02266-4.

## Supplementary information

Reporting Summary

Supplementary Video 1 Example of a recording of the LPtA of a WT mouse injected with the BDNF–mScarlet AAV in the V2mm 21 days after injection. The video is played back at 20 fps (13×); scale bar, 15 μm.

Supplementary Video 2 Supplementary Video 1 was tracked using the ImageJ plugin Trackmate and manual tracking. The tracks obtained are shown in overlay. The video is played back at 20 fps (13×); scale bar, 15 μm.

## Source data

Source Data Fig. 2 Statistical source data and unprocessed western blots.

Source Data Fig. 3 Statistical source data and unprocessed western blots.

Source Data Fig. 4 Statistical source data.

Source Data Fig. 5 Statistical source data.

Source Data Fig. 6 Statistical source data.

Source Data Fig. 7 Statistical source data.

Source Data Extended Data Figs. 1 and 2 Unprocessed western blots.

Source Data Extended Data Fig. 1 Statistical source data.

Source Data Extended Data Fig. 2 Statistical source data.

Source Data Extended Data Fig. 3 Statistical source data.

Source Data Extended Data Fig. 4 Statistical source data.

Source Data Extended Data Fig. 5 Statistical source data.

Source Data Extended Data Fig. 6 Statistical source data.

Source Data Extended Data Fig. 7 Statistical source data.

## Data Availability

Source data are provided with this paper. Other data are available upon request
