# Supplementary material for: Aberrant tau accumulation caused by MAPT mutations induces early pathological changes in axonal transport that are rescued by p38α inhibition
Source: Nat Neurosci. 2026 Jun 4;29(6):1355–68. doi: 10.1038/s41593-026-02266-4 (PMC13246509; doi:10.1038/s41593-026-02266-4)
Supplement: Supplementary file 1 — Reporting Summary [file 41593_2026_2266_MOESM1_ESM.pdf]

nature portfolio

Corresponding author(s): Edoardo Moretto, Giampietro Schiavo

Last updated by author(s): Jan 15, 2026

Reporting Summary

Nature Portfolio wishes to improve the reproducibility of the work that we publish. This form provides structure for consistency and transparency in reporting. For further information on Nature Portfolio policies, see our Editorial Policies and the Editorial Policy Checklist.

Statistics

For all statistical analyses, confirm that the following items are present in the figure legend, table legend, main text, or Methods section.

n/a	Confirmed	
☐	☒	The exact sample size (*n*) for each experimental group/condition, given as a discrete number and unit of measurement
☐	☒	A statement on whether measurements were taken from distinct samples or whether the same sample was measured repeatedly
☐	☒	The statistical test(s) used AND whether they are one- or two-sided<br>*Only common tests should be described solely by name; describe more complex techniques in the Methods section.*
☒	☐	A description of all covariates tested
☐	☒	A description of any assumptions or corrections, such as tests of normality and adjustment for multiple comparisons
☐	☒	A full description of the statistical parameters including central tendency (e.g. means) or other basic estimates (e.g. regression coefficient) AND variation (e.g. standard deviation) or associated estimates of uncertainty (e.g. confidence intervals)
☐	☒	For null hypothesis testing, the test statistic (e.g. F, t, r) with confidence intervals, effect sizes, degrees of freedom and P value noted<br>*Give P values as exact values whenever suitable.*
☒	☐	For Bayesian analysis, information on the choice of priors and Markov chain Monte Carlo settings
☒	☐	For hierarchical and complex designs, identification of the appropriate level for tests and full reporting of outcomes
☒	☐	Estimates of effect sizes (e.g. Cohen's *d*, Pearson's *r*), indicating how they were calculated

Our web collection on statistics for biologists contains articles on many of the points above.

Software and code

Policy information about availability of computer code

Data collection	Softwares used are described in the method section.
Data analysis	Softwares used are described in the method section.

For manuscripts utilizing custom algorithms or software that are central to the research but not yet described in published literature, software must be made available to editors and reviewers. We strongly encourage code deposition in a community repository (e.g. GitHub). See the Nature Portfolio guidelines for submitting code & software for further information.

Data

Policy information about availability of data

All manuscripts must include a data availability statement. This statement should provide the following information, where applicable:

- Accession codes, unique identifiers, or web links for publicly available datasets
- A description of any restrictions on data availability
- For clinical datasets or third party data, please ensure that the statement adheres to our policy

Data are available upon reasonable request

Research involving human participants, their data, or biological material

Policy information about studies with human participants or human data. See also policy information about sex, gender (identity/presentation), and sexual orientation and race, ethnicity and racism.

Reporting on sex and gender	*Use the terms sex (biological attribute) and gender (shaped by social and cultural circumstances) carefully in order to avoid confusing both terms. Indicate if findings apply to only one sex or gender; describe whether sex and gender were considered in study design; whether sex and/or gender was determined based on self-reporting or assigned and methods used. Provide in the source data disaggregated sex and gender data, where this information has been collected, and if consent has been obtained for sharing of individual-level data; provide overall numbers in this Reporting Summary. Please state if this information has not been collected. Report sex- and gender-based analyses where performed, justify reasons for lack of sex- and gender-based analysis.*
Reporting on race, ethnicity, or other socially relevant groupings	*Please specify the socially constructed or socially relevant categorization variable(s) used in your manuscript and explain why they were used. Please note that such variables should not be used as proxies for other socially constructed/relevant variables (for example, race or ethnicity should not be used as a proxy for socioeconomic status). Provide clear definitions of the relevant terms used, how they were provided (by the participants/respondents, the researchers, or third parties), and the method(s) used to classify people into the different categories (e.g. self-report, census or administrative data, social media data, etc.) Please provide details about how you controlled for confounding variables in your analyses.*
Population characteristics	*Describe the covariate-relevant population characteristics of the human research participants (e.g. age, genotypic information, past and current diagnosis and treatment categories). If you filled out the behavioural & social sciences study design questions and have nothing to add here, write "See above."*
Recruitment	*Describe how participants were recruited. Outline any potential self-selection bias or other biases that may be present and how these are likely to impact results.*
Ethics oversight	*Identify the organization(s) that approved the study protocol.*

Note that full information on the approval of the study protocol must also be provided in the manuscript.

Field-specific reporting

Please select the one below that is the best fit for your research. If you are not sure, read the appropriate sections before making your selection.

☒ Life sciences ☐ Behavioural & social sciences ☐ Ecological, evolutionary & environmental sciences

For a reference copy of the document with all sections, see nature.com/documents/nr-reporting-summary-flat.pdf

Life sciences study design

All studies must disclose on these points even when the disclosure is negative.

Sample size	Sample sizes were determined based on standard practice in the literature.
Data exclusions	Data exclusion criteria are specified in the method section, when applicable.
Replication	Experiments were replicated a standard number of times (indicated in each figure legend) based on normal practices in the field.
Randomization	Mice and neuronal cultures were randomly allocated to each experimental group.
Blinding	Data analysis was performed in a blinded manner.

Reporting for specific materials, systems and methods

We require information from authors about some types of materials, experimental systems and methods used in many studies. Here, indicate whether each material, system or method listed is relevant to your study. If you are not sure if a list item applies to your research, read the appropriate section before selecting a response.

Materials & experimental systems

n/a	Involved in the study
☐	☒ Antibodies
☐	☒ Eukaryotic cell lines
☒	☐ Palaeontology and archaeology
☐	☒ Animals and other organisms
☒	☐ Clinical data
☒	☐ Dual use research of concern
☒	☐ Plants

Methods

n/a	Involved in the study
☒	☐ ChIP-seq
☒	☐ Flow cytometry
☒	☐ MRI-based neuroimaging

Antibodies

Antibodies used

Antibodies used are described in the method section.

Validation

α-p38α, Cell signalling # 9218. Validated against other isoforms of p38 in https://www.cellsignal.com/products/primary-antibodies/p38a-mapk-antibody/9218 and in https://www.ncbi.nlm.nih.gov/pmc/articles/PMC5964181/
α-p-p38, Cell Signalling #4511. Validated in https://www.cellsignal.com/products/primary-antibodies/phospho-p38-mapk-thr180-tyr182-d3f9-xp-rabbit-mab/4511

α-GAPDH, Abcam ab245355. https://www.abcam.com/en-it/products/primary-antibodies/gapdh-antibody-ab245355#application=wb. Used in at least 27 manuscripts according to CiteAb
α-hTau, Biolegend, 15-25 Antibody TAU13. The same clone has been validated in https://www.biorxiv.org/content/10.1101/2023.04.13.536711v1.full

α-AT8, ThermoFisher Scientific #MN1020. Widespread use in the literature. Validated in https://www.biorxiv.org/content/10.1101/2023.04.13.536711v1.full

α-FLAG, M2 F1804, Sigma Aldrich. Widespread use in the literature, more than 8000 citations according to CiteAb

α-β3 tubulin, Biolegend clone TU27/Tubulin. This has been mistakenly reported in the manuscript. The correct one is Biolegend, Covance Catalog# MMS-435P. Used in more than 750 publications according to Biolegend. https://www.biolegend.com/en-gb/products/purified-anti-tubulin-beta-3-tubb3-antibody-11580

α-tau, Dako A0024. Validated in https://www.biorxiv.org/content/10.1101/2023.04.13.536711v1.full

α-GFP, Aves GFP-1020. Used in more than 2000 publications according to https://www.aveslabs.com/products/anti-green-fluorescent-protein-antibody-gfp

α-mScarlet, Synaptic System N1302-AF568-L. Validate in https://nano-tag.com/product/fluotag-x2-anti-mscarlet-i/

α-NeuN, Abcam ab128886. Validated in https://www.abcam.com/en-it/products/primary-antibodies/neun-antibody-neuronal-marker-ab128886#

α-Alz50. Produced in house from the hybridoma cell line ATCC-HB-9205, https://www.alzforum.org/antibodies/tau-alz50-0

Eukaryotic cell lines

Policy information about cell lines and Sex and Gender in Research

Cell line source(s)

Lenti-X HEK293 cells used for lentiviral production are available from Takara Clontech.
The sex of primary neurons was not checked as these are prepared mixing tissue from several embryos of the same litter.

Authentication

No further authentication was performed after purchase of the Lenti X HEK 293.
The neuronal nature of the cells analysed in experiments using neuronal cultures or in vivo was based on the use of neuronal specific promoters for expression of the genes of interest as described in the manuscript.

Mycoplasma contamination

Lenti X HEK 293 cells were routinely tested for mycoplasma.

Commonly misidentified lines
(See ICLAC register)

Name any commonly misidentified cell lines used in the study and provide a rationale for their use.

Animals and other research organisms

Policy information about studies involving animals; ARRIVE guidelines recommended for reporting animal research, and Sex and Gender in Research

Laboratory animals

For the preparation of neuronal primary cultures, we used E16 C57/BL6 embryos.

Laboratory animals

In vivo experiments were performed on two separate strains of Mus musculus, rTg4510 and rTg21221. Age and sex of the animals used are described in each figure legend. The breeding scheme is described in the method section.

Wild animals

Provide details on animals observed in or captured in the field; report species and age where possible. Describe how animals were caught and transported and what happened to captive animals after the study (if killed, explain why and describe method; if released, say where and when) OR state that the study did not involve wild animals.

Reporting on sex

The sex of the animals used in the different experiments is described in the figure legends.

Field-collected samples

For laboratory work with field-collected samples, describe all relevant parameters such as housing, maintenance, temperature, photoperiod and end-of-experiment protocol OR state that the study did not involve samples collected from the field.

Ethics oversight

Experiments involving mice were performed under license from the UK Home Office in accordance with the Animals (Scientific Procedures) Act (1986) and were approved by the UCL Queen Square Institute of Neurology Ethical Review Committee.

Note that full information on the approval of the study protocol must also be provided in the manuscript.

Plants

Seed stocks

Report on the source of all seed stocks or other plant material used. If applicable, state the seed stock centre and catalogue number. If plant specimens were collected from the field, describe the collection location, date and sampling procedures.

Novel plant genotypes

Describe the methods by which all novel plant genotypes were produced. This includes those generated by transgenic approaches, gene editing, chemical/radiation-based mutagenesis and hybridization. For transgenic lines, describe the transformation method, the number of independent lines analyzed and the generation upon which experiments were performed. For gene-edited lines, describe the editor used, the endogenous sequence targeted for editing, the targeting guide RNA sequence (if applicable) and how the editor was applied.

Authentication

Describe any authentication procedures for each seed stock used or novel genotype generated. Describe any experiments used to assess the effect of a mutation and, where applicable, how potential secondary effects (e.g. second site T-DNA insertions, mosiacism, off-target gene editing) were examined.
