# Supplementary material for: Aberrant tau accumulation caused by MAPT mutations induces early pathological changes in axonal transport that are rescued by p38α inhibition
Source: Nat Neurosci. 2026 Jun 4;29(6):1355–68. doi: 10.1038/s41593-026-02266-4 (PMC13246509; doi:10.1038/s41593-026-02266-4)

Extended data Fig. 1e uncropped unprocessed blots

pMNK1

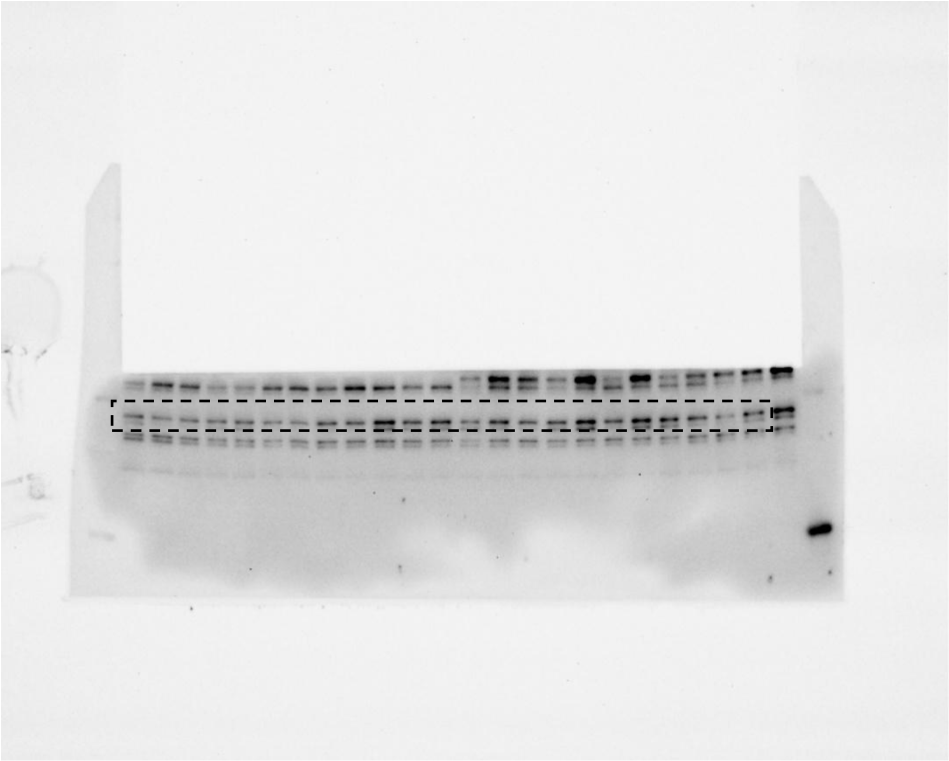

Coomassie pMNK1

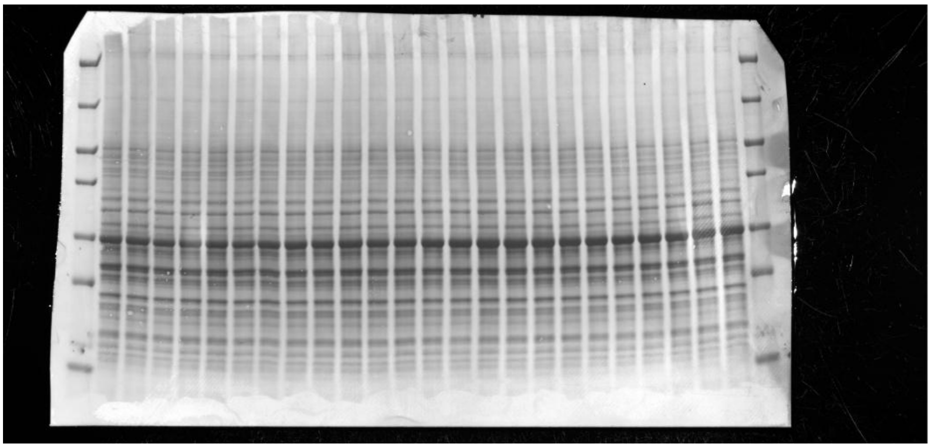

MNK1

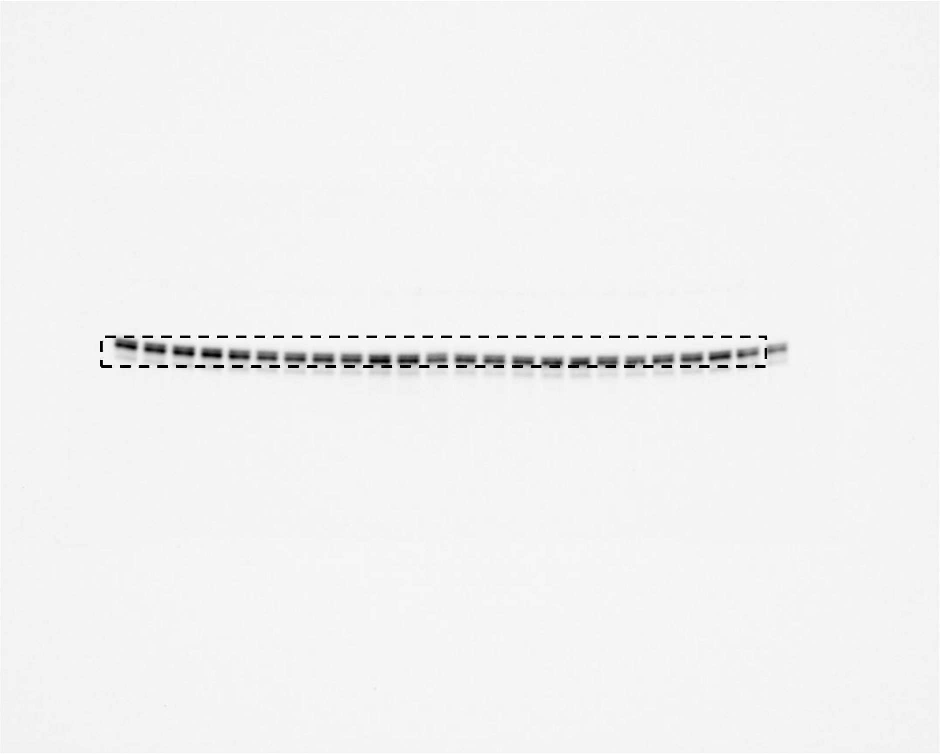

Coomassie MNK1

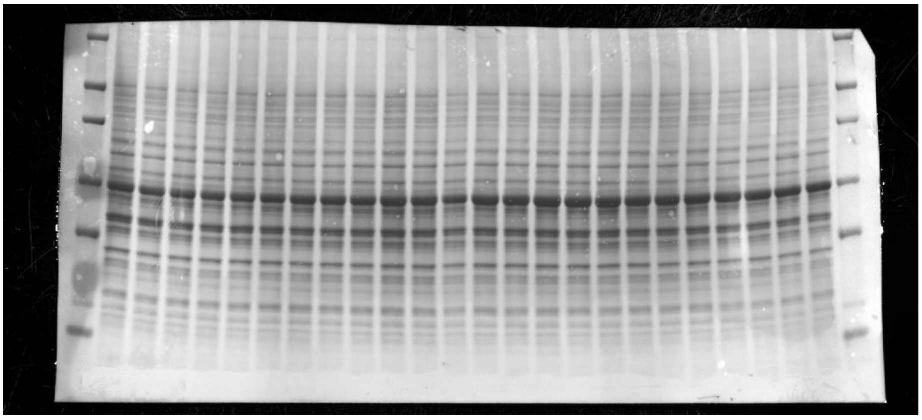

Extended data Fig. 2e uncropped unprocessed blots

p-p38

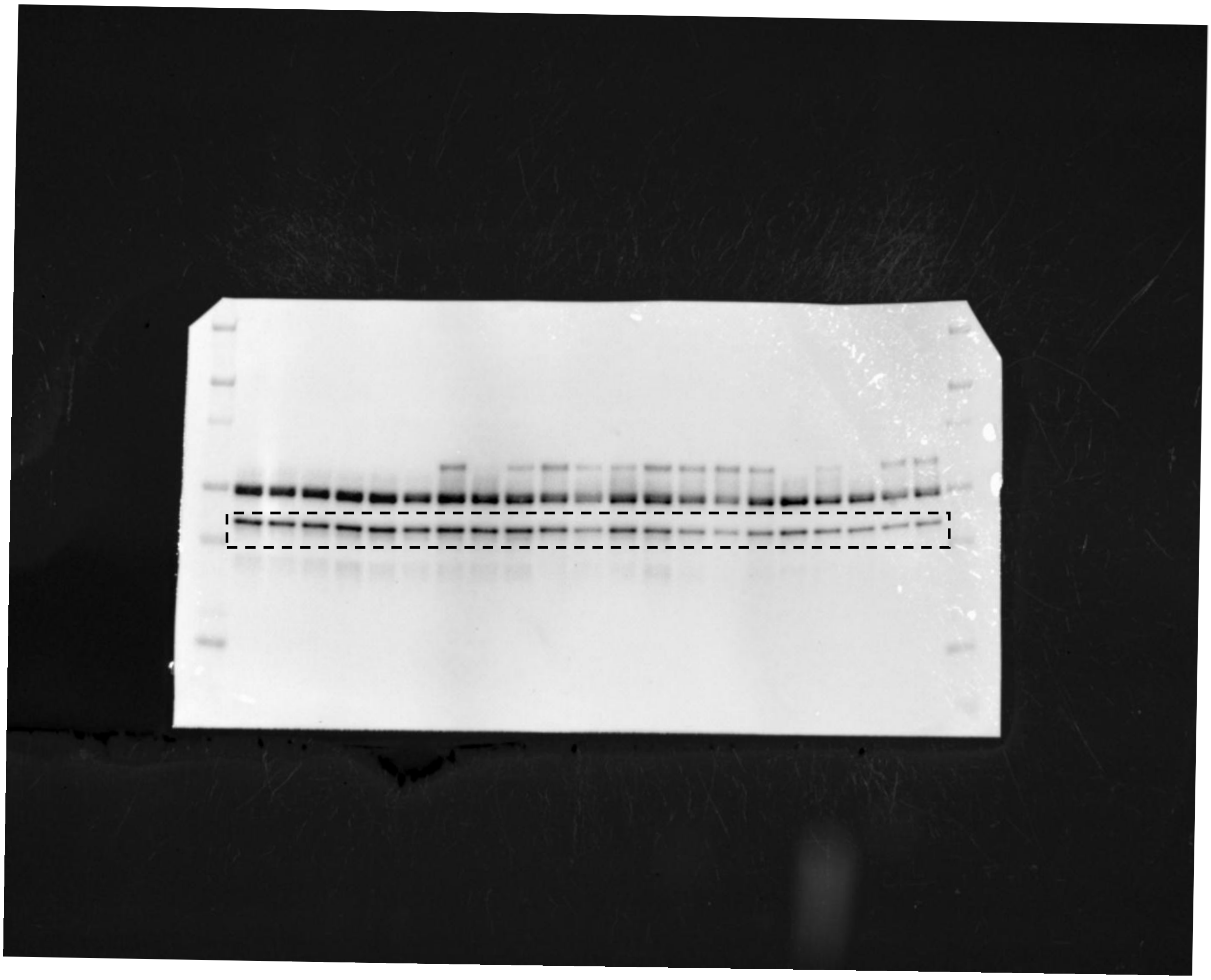

Coomassie p-p38

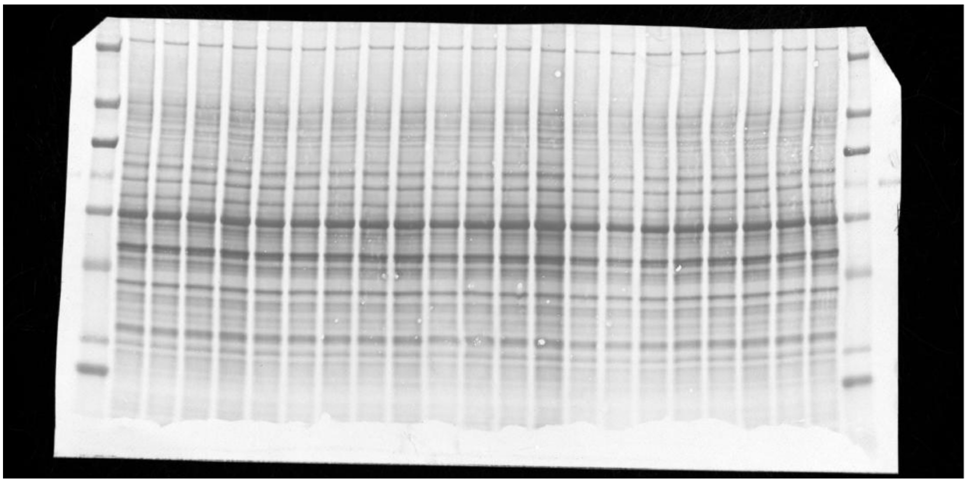

p38α

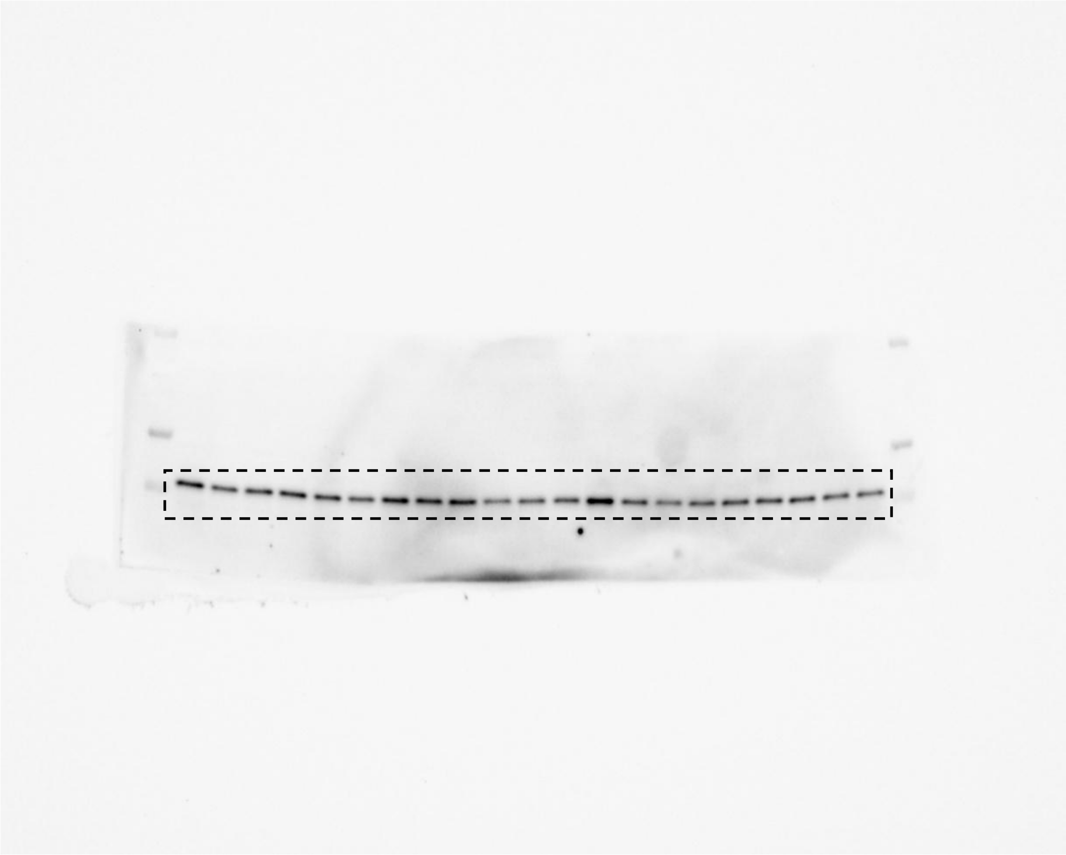

Coomassie p38α

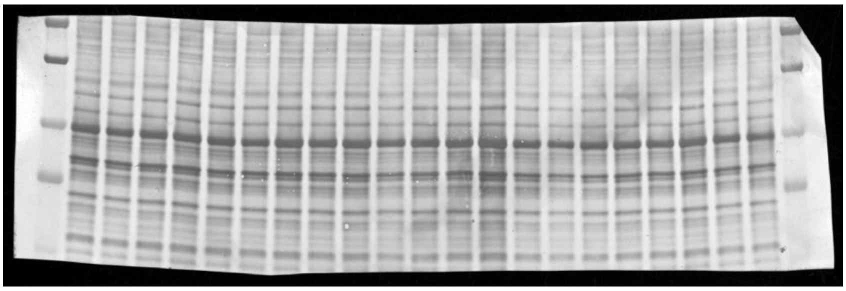

AT8

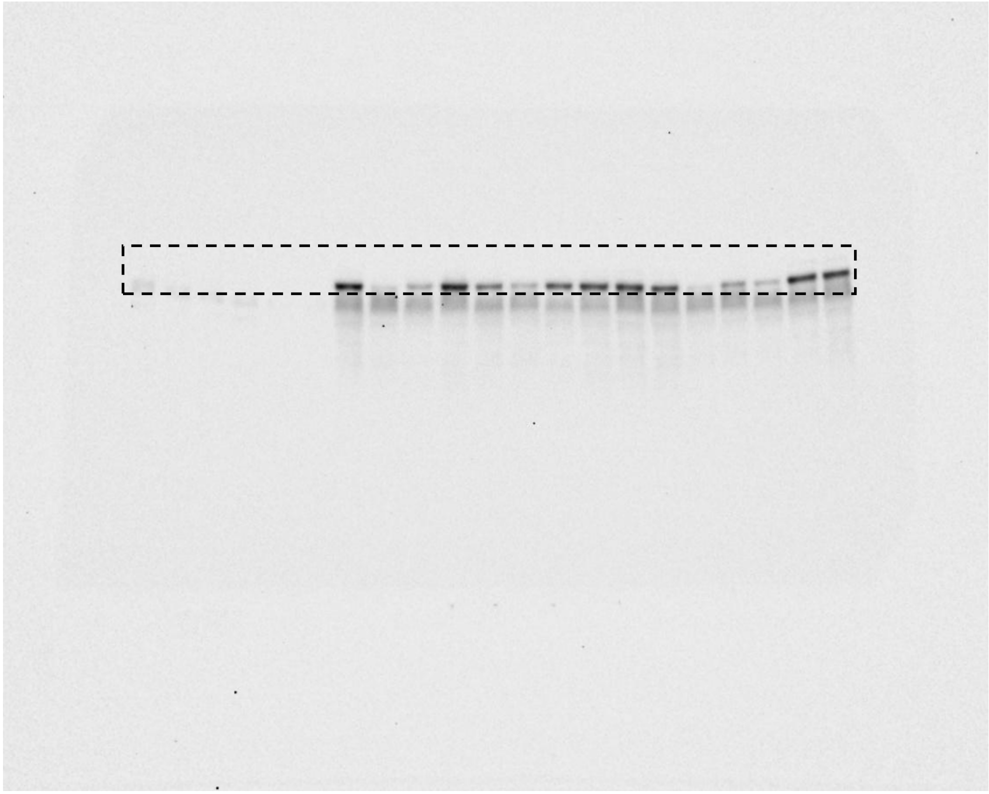

Coomassie AT8

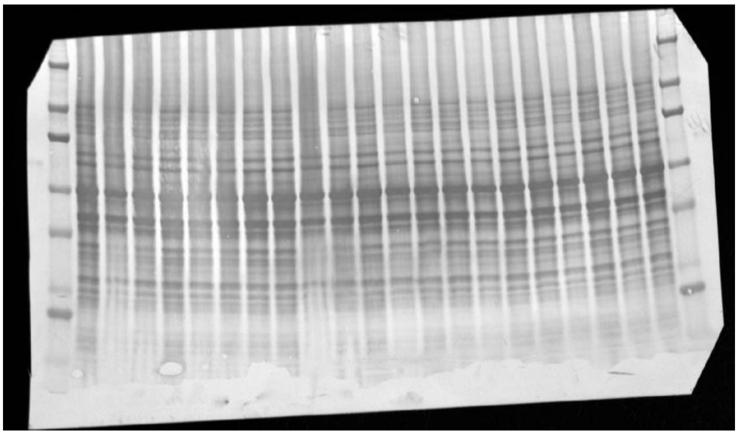

DA9

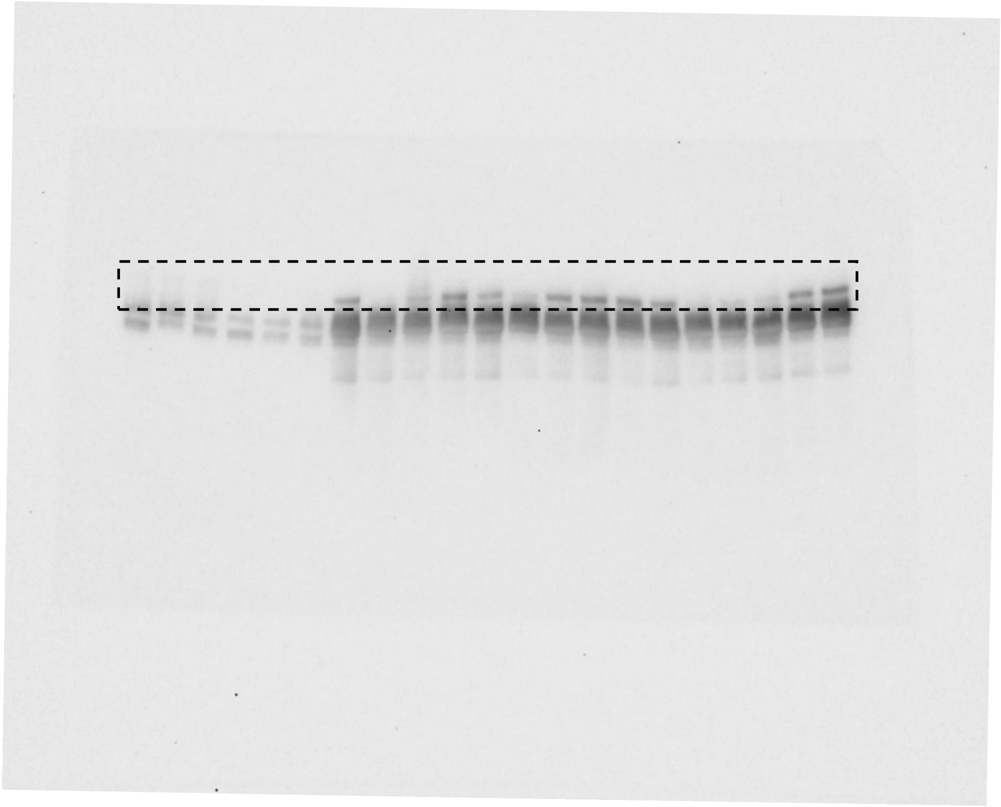

Coomassie DA9

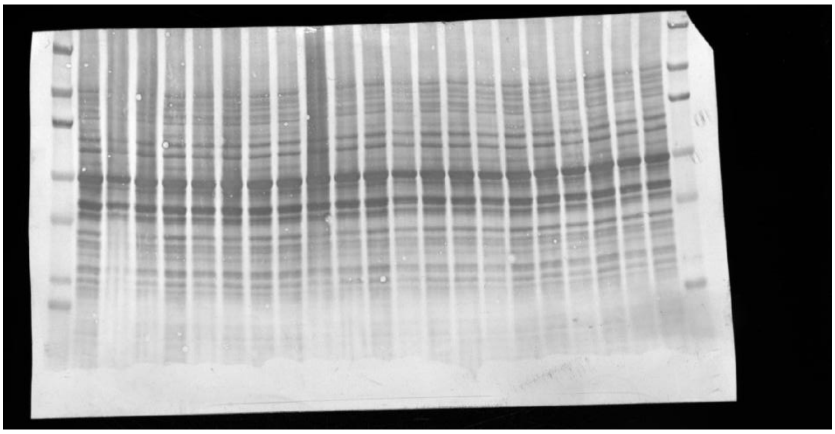

Supplement: Supplementary file 10 — Unprocessed western blots. [file 41593_2026_2266_MOESM10_ESM.pdf]
